# Supplementary figures and images for: Conservation of heat stress acclimation by the IPK2-type kinases that control the synthesis of the inositol pyrophosphate 4/6-InsP7 in land plants
Source: PLoS Genet. 2025 Sep 11;21(9):e1011838. doi: 10.1371/journal.pgen.1011838 (PMC12445474; doi:10.1371/journal.pgen.1011838)

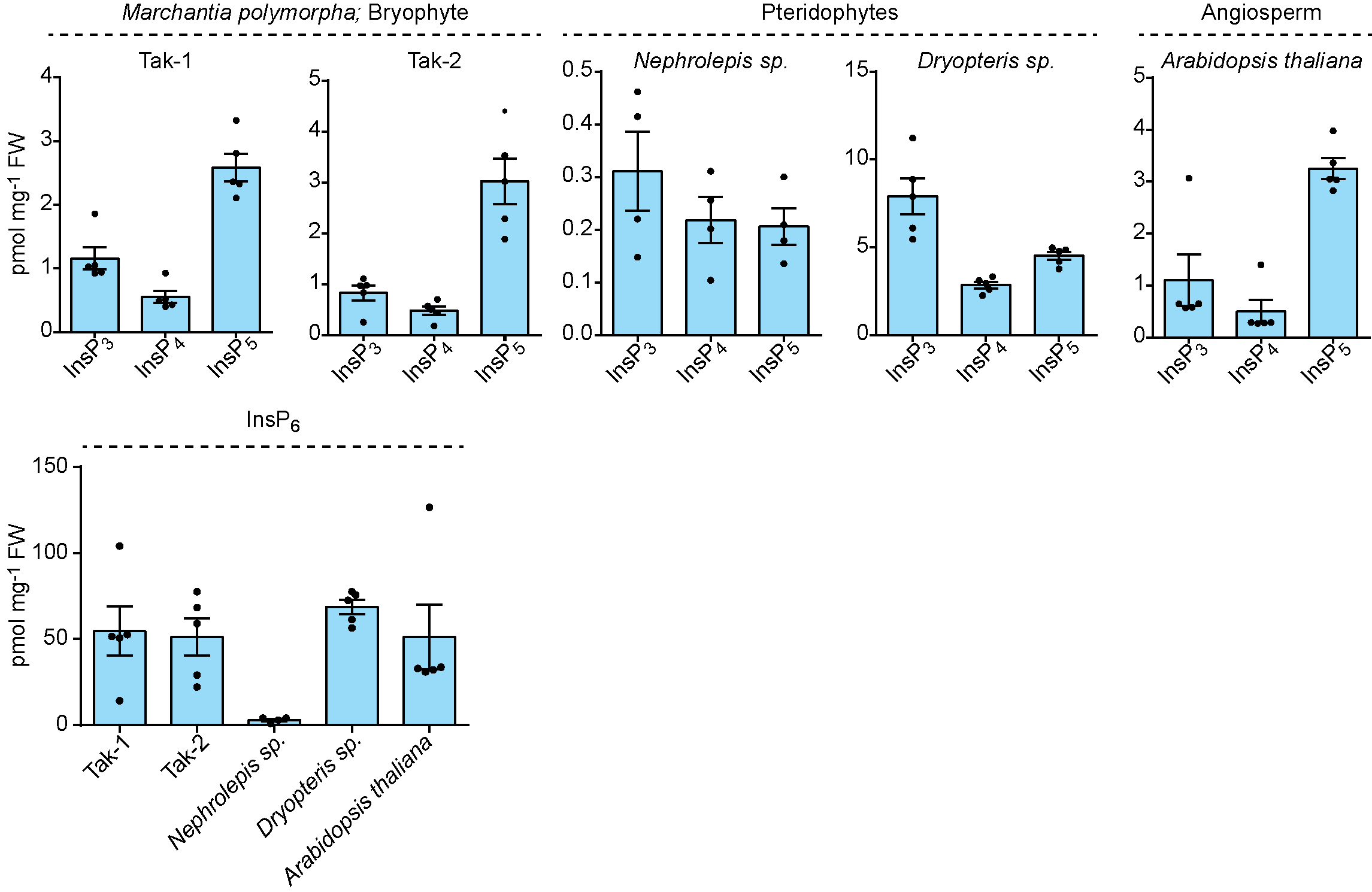

Supplement: S1 Fig — Quantification of different inositol phosphates detected in the above-mentioned embryophytes through CE-MS. Purified InsP extracts of 14-day-old M. polymorpha thalli (Tak-1 and Tak-2), mature sporophylls of pteridophytes (Nephrolepis sp. and Dryopteris sp.) and 14-day-old A. thaliana (Col-0) seedlings were subjected to CE-MS. The InsP5 species were assigned by mass spectrometry and identical migration time compared with relative standards. Data are means ± SEM (n ≥ 4 biological replicates). (TIF) [file pgen.1011838.s001.tif]

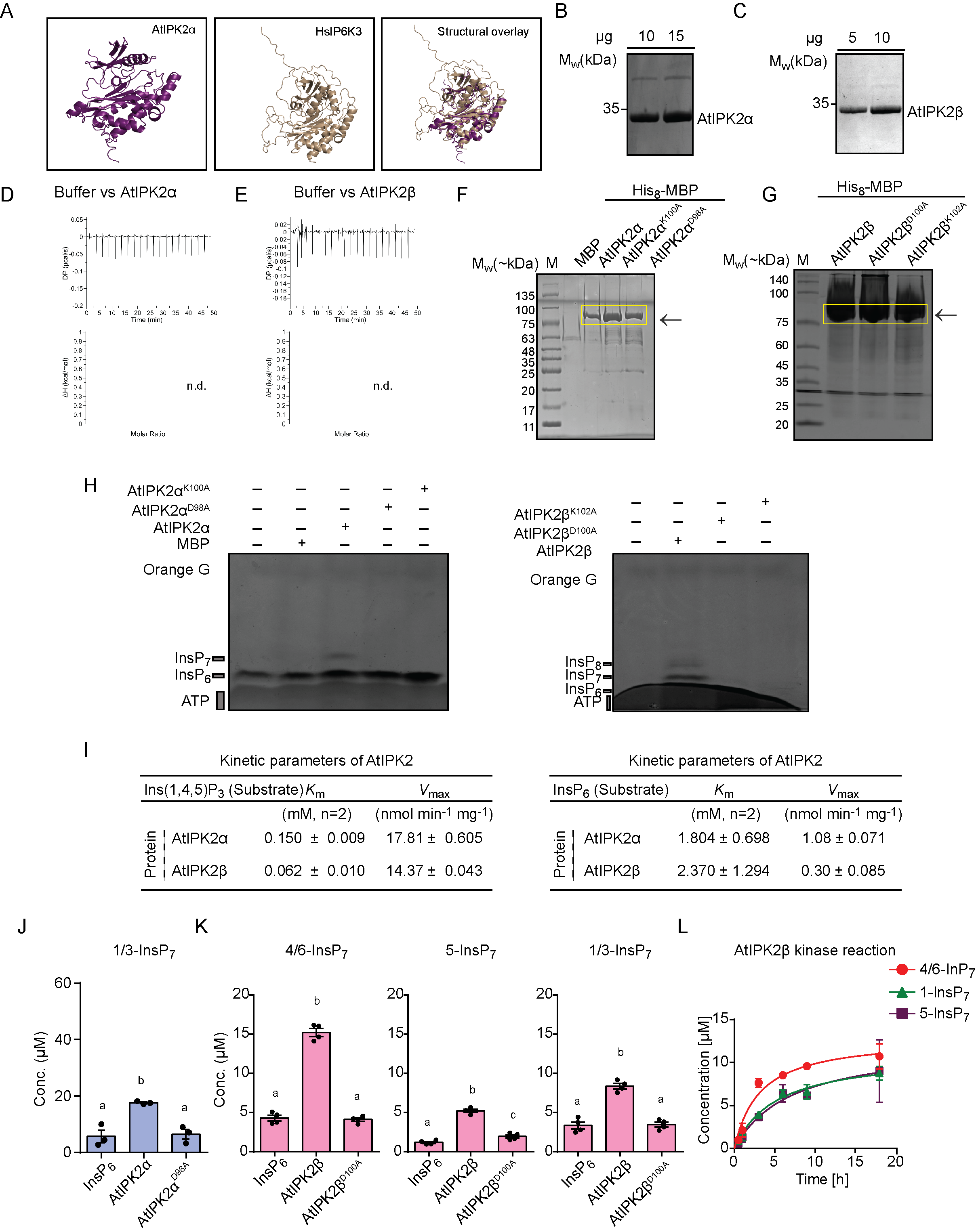

Supplement: S2 Fig — A. Structural models (overview) of AtIPK2α (Protein Data Bank entry 4FRF) (pink) and HsIP6K3 (golden). Models were obtained by the AlphaFold web portal (https://alphafold.ebi.ac.uk/) and built on the Pymol. Overlay of AtIPK2α (hot pink) and HsIP6K3 (golden) structures (RMSD value = 0.958). B and C. SDS-PAGE analyses of tag-free recombinant AtIPK2α and AtIPK2β proteins used for ITC experiments. Recombinant His8-MBP-TEV-AtIPK2 proteins were subjected to TEV protease and the digested products were further purified using affinity-based chromatography. The tag-free proteins were loaded on gel. Resolved proteins were visualized by coomassie blue staining. D and E. Isothermal titration calorimetry (ITC) assays of AtIPK2α (10 µM; left panel) and AtIPK2β (10 µM; right panel) (in cell) with ITC buffer (in syringe), respectively. Raw heats per injection are shown in the top panel and the bottom panel represents the integrated heats of each injection. F and G. SDS-PAGE analysis of the recombinant AtIPK2α and its catalytic dead variants protein in translational fusion with N-terminal His8-MBP-TEV tag. Resolved proteins were visualized by coomassie blue staining (F). SDS-PAGE analysis of AtIPK2β WT and the catalytic dead variants protein. Resolved proteins were visualized by coomassie blue staining (G). H. PAGE analysis of in vitro kinase assay reaction products of AtIPK2s. InsP6 alone and MBP served as control. I. Table showing the kinetic parameters (Km and Vmax) of AtIPK2α and AtIPK2β for Ins(1,4,5)P3 and InsP6 at varying ATP concentration. Km and Vmax were obtained after fitting of the data against the Michaelis-Menten model. J. Quantification of the AtIPK2α reaction product using CE-MS analyses. A minor amount of 1/3-InsP7 species could be detected in the reaction products. Data represent means ± SEM (n = 3). Letters depict the significance in one-way ANOVA followed by Dunnett’s test (a and b, P < 0.0001; a and c, P < 0.05). K. AtIPK2β phosphorylates InsP6 to synthesize 4/6- [file pgen.1011838.s002.tif]

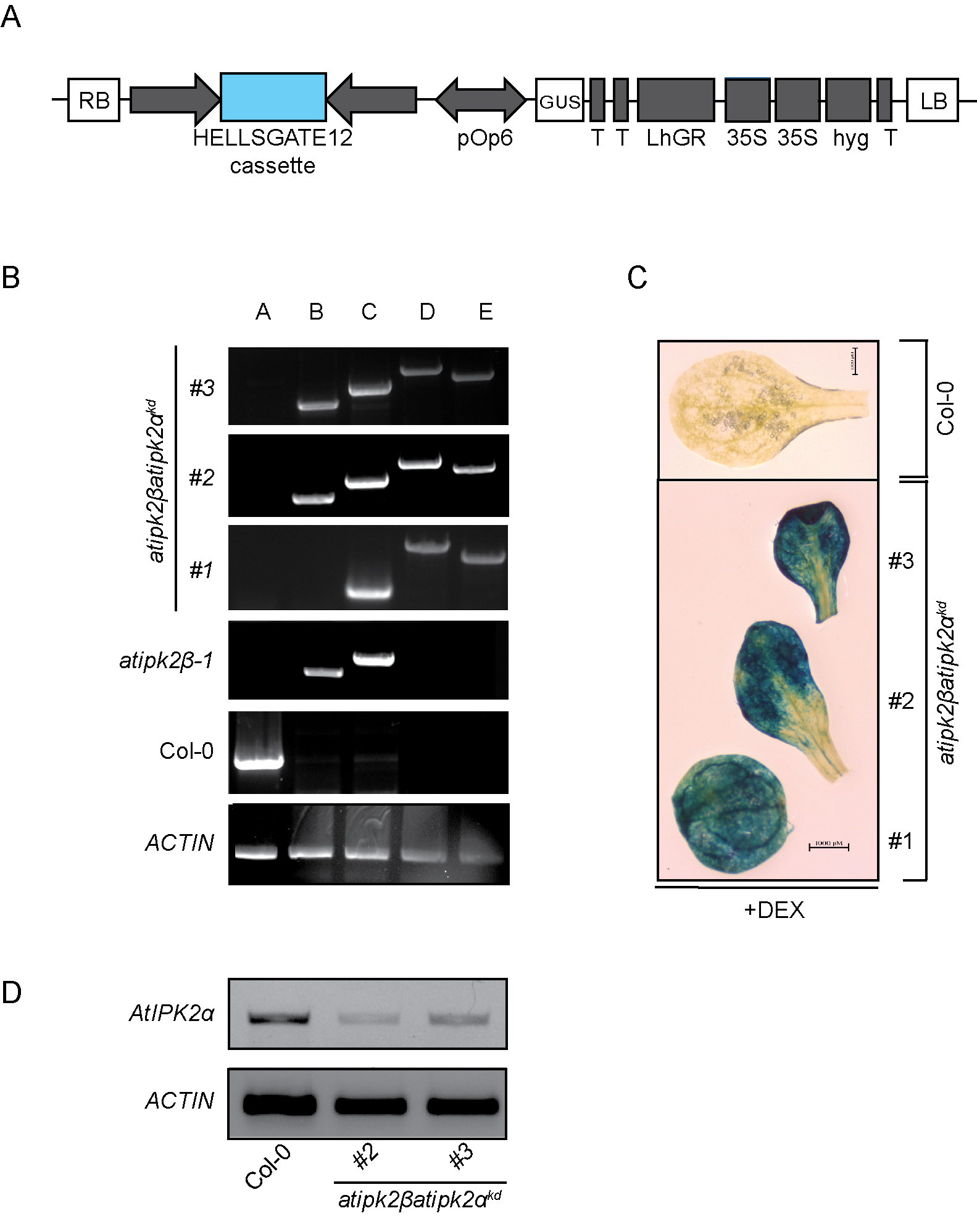

Supplement: S3 Fig — A. Schematic diagram of the pOpOff2 vector. RB, right border; T, terminator; hyg, hygromycin; LB, left border. B. Genotyping PCR of Col-0, atipk2β-1 and all the three atipk2α knockdown lines. A to E represents the primers set used for genotyping, details of the primers are mentioned S1 Table. ACTIN served as a reference gene. C. Representative images of the GUS signal in leaves of Col-0 and the three independent atipk2βatipk2αkd lines used in this study after dexamethasone (DEX) treatment. D. Stability of AtIPK2α transcript is affected in the atipk2βatipk2αkd lines. Expression analyses of AtIPK2α between Col-0 and independent atipk2βatipk2αkd lines using RT-PCR. ACTIN served as reference gene. (TIF) [file pgen.1011838.s003.tif]

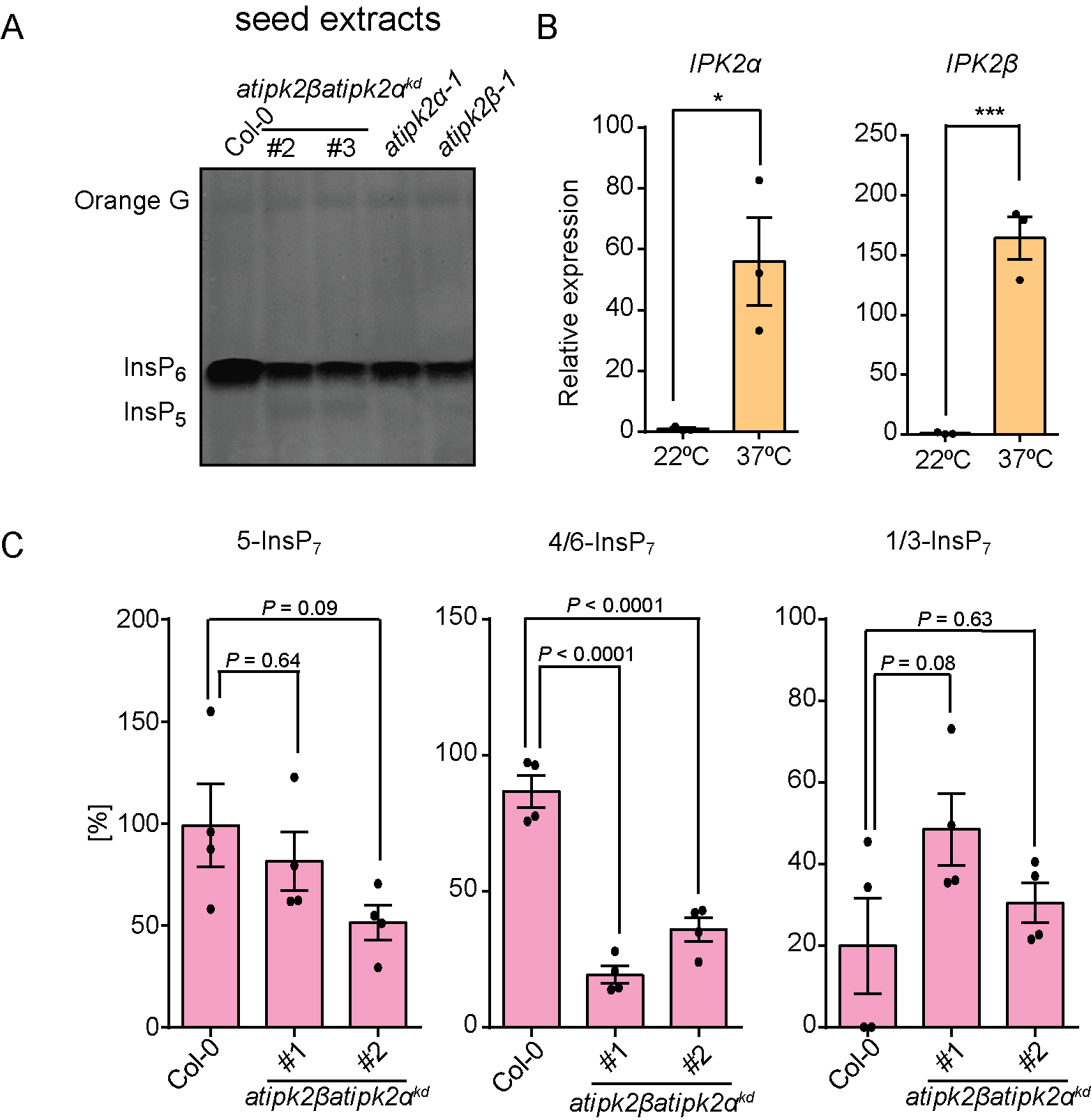

Supplement: S4 Fig — A. PAGE analysis of seed extracts of Col-0, atipk2βatipk2αkd, atipk2α-1 and atipk2β-1 lines. This result is in agreement with the previously published report [36,50,63] that AtIPK2 contributes to InsP homeostasis distinctively in different plant parts. B. Quantitative RT-PCR (qRT-PCR) analysis of AtIPK2α and AtIPK2β in Col-0 after heat shock. 14-day-old seedlings were exposed to 37⁰C for 3 h and were harvested for qRT-PCR analysis. TUBULIN was used as a reference gene. Values are means ± SEM (n = 3, biological replicates). C. CE-MS analyses of InsP extracts of Col-0 and atipk2βatipk2αkd seedlings after heat shock of 3 h at 37⁰C. Graph represents the fold difference of InsP isomers of the designated genotypes upon heat stress. Values are ± SEM (n = 4, biological replicates). Statistical significance is determined in one-way ANOVA followed by Dunnett’s test. (TIF) [file pgen.1011838.s004.tif]

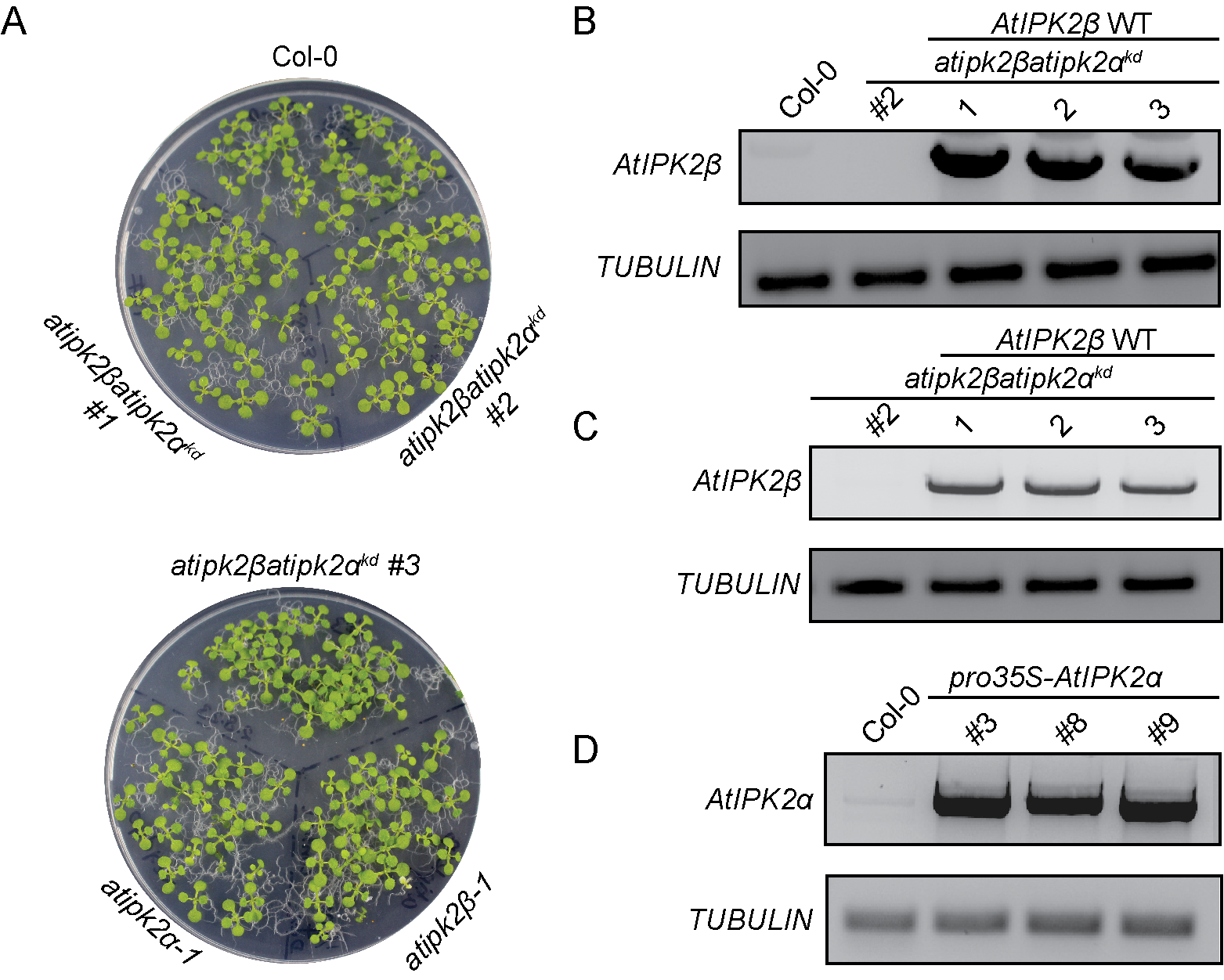

Supplement: S5 Fig — A. Photograph of the control plate maintained at 22⁰C throughout basal thermal tolerance assay. This is the control set for the experiment presented in the main Fig 4C. B. Genotyping PCR of atipk2βatipk2αkd lines expressing AtIPK2β under the control of a constitutive 35S promoter. The primers used for genotyping are mentioned in S1 Table. TUBULIN served as a reference gene. C. Expression analyses of AtIPK2β using RT-PCR. The primers used for RT-PCR are mentioned in S1 Table. TUBULIN served as a reference gene. D. Genotyping PCR of AtIPK2α in pro35S::AtIPK2α overexpression lines. The primers used for genotyping PCR are mentioned in S1 Table. TUBULIN served as a reference gene. (TIF) [file pgen.1011838.s005.tif]

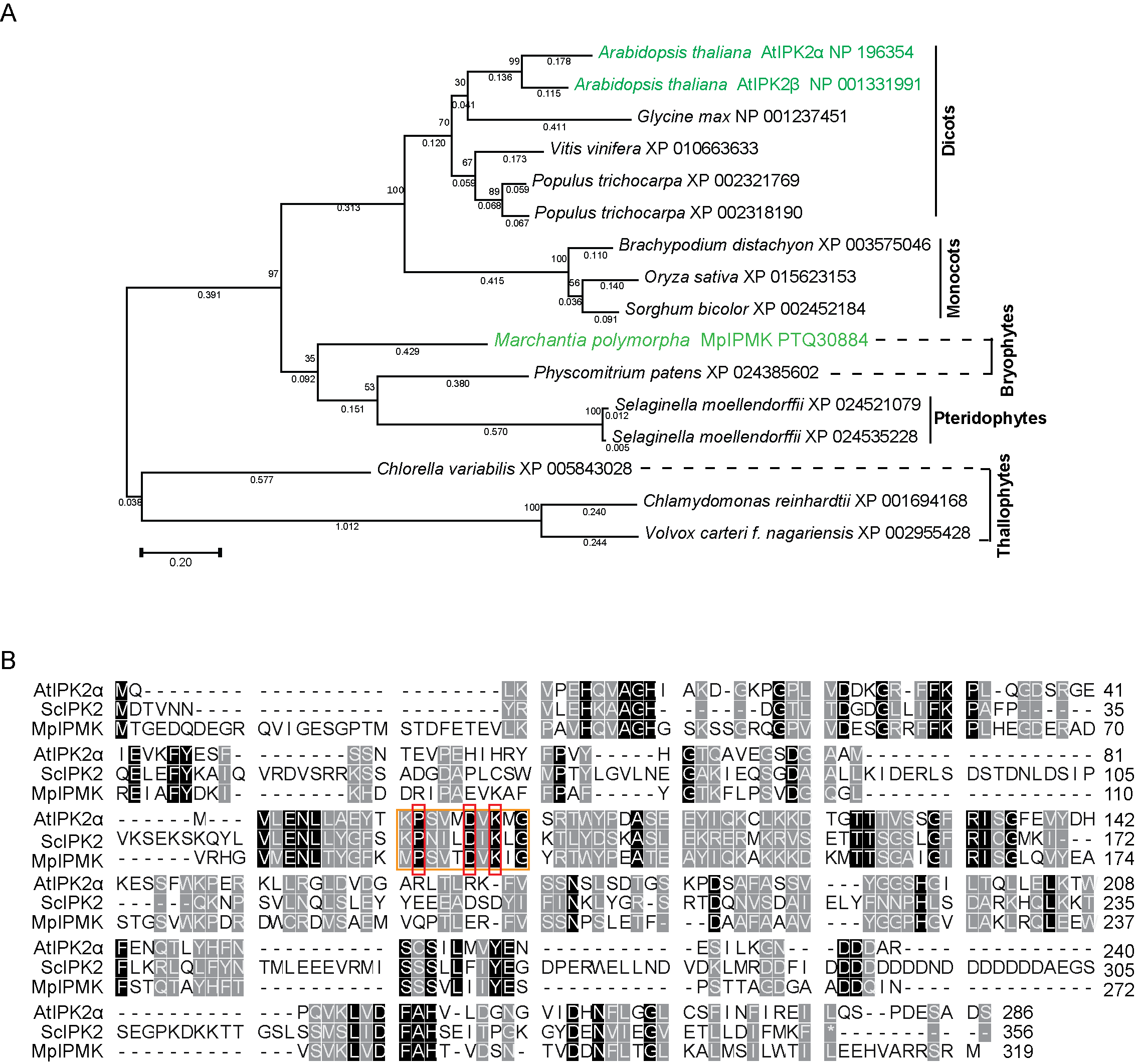

Supplement: S6 Fig — A. The phylogenetic tree was estimated from an alignment of AtIPK2α amino acid sequences using maximum likelihood. Branch support was calculated from 1000 bootstrap replicates, and values below 50% are omitted. Branch lengths are given in terms of expected numbers of amino acid substitutions per site. B. Protein alignment of MpIPMK with AtIPK2α and ScIpk2. Red rectangle marks the conserved catalytic motif PXXXDXKXG of the InsP kinase. (TIF) [file pgen.1011838.s006.tif]

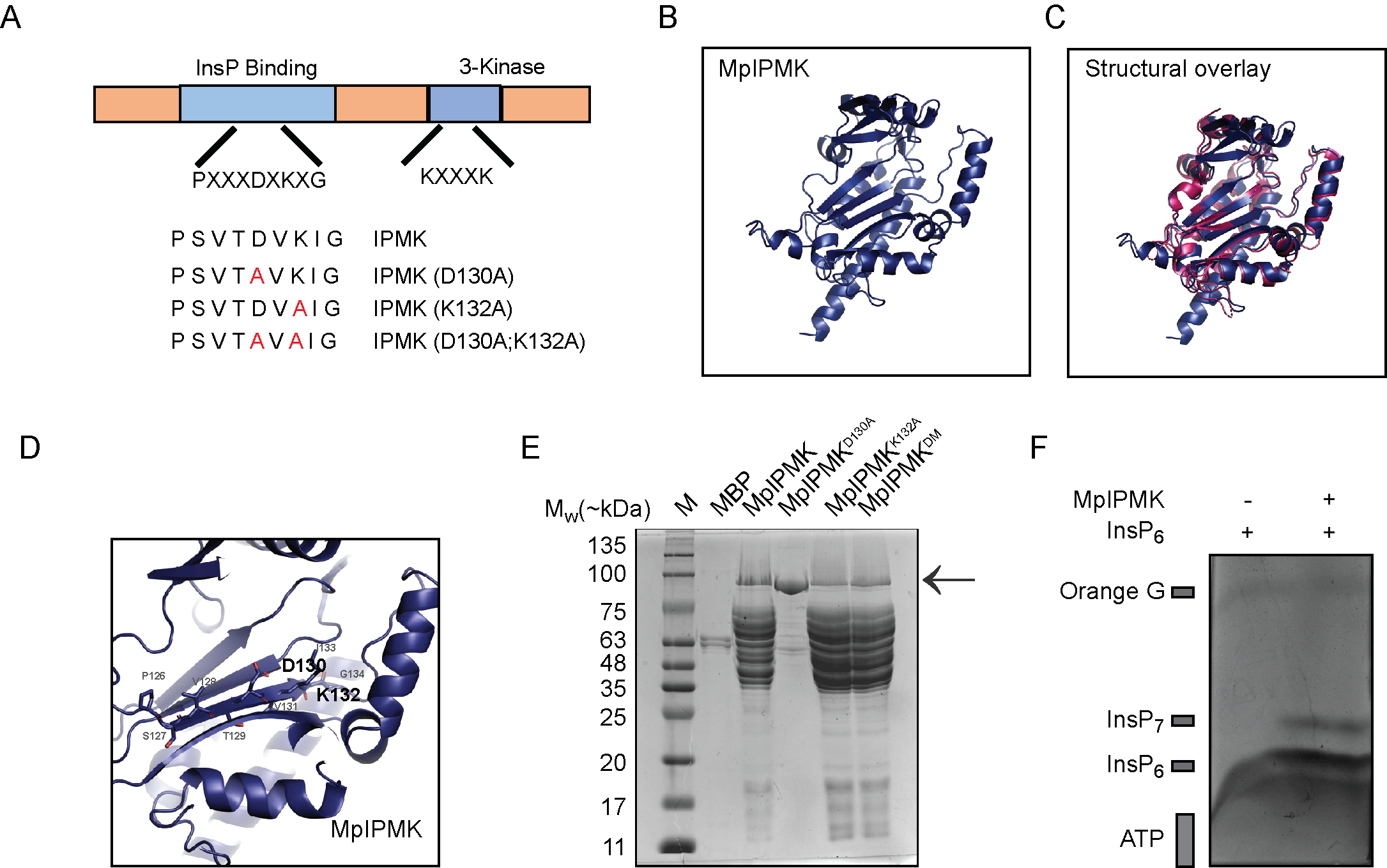

Supplement: S7 Fig — A. Cartoon depicting the conserved PXXXDXKXG motif of M. polymorpha IPMK. The residues highlighted in red are the altered residues, forming catalytic dead variants of IPMK, i.e., MpIPMKD130A, MpIPMKK132A, MpIPMKD130AK132A (referred as MpIPMKDM). B. Structural model (Structural model (overview) of MpIPMK. Models were obtained by the AlphaFold web portal (https://alphafold.ebi.ac.uk/) and built on the Pymol. C. Structural overlay of AtIPK2α (hot pink), MpIPMK (blue) structures (RMSD value = 0.872). Note the similarity between the MpIPMK model and the AtIPK2α structure (Protein Data Bank entry 4FRF). D. Zoom-in-into view of the catalytic active site of MpIPMK. E. SDS-PAGE analysis of MpIPMK and its catalytic dead variants. Arrow head denotes MpIPMK and its catalytic dead variants. F. PAGE analysis of the in vitro kinase reaction products of MpIPMK. Recombinant His8-MBP-MpIPMK was incubated with 12.5 mM ATP, and 10 nmol InsP6 at 37⁰C for 12 h in reaction buffer. The reaction product was separated by 33% PAGE and visualized with toluidine blue. InsP6 alone served as a control. (TIF) [file pgen.1011838.s007.tif]

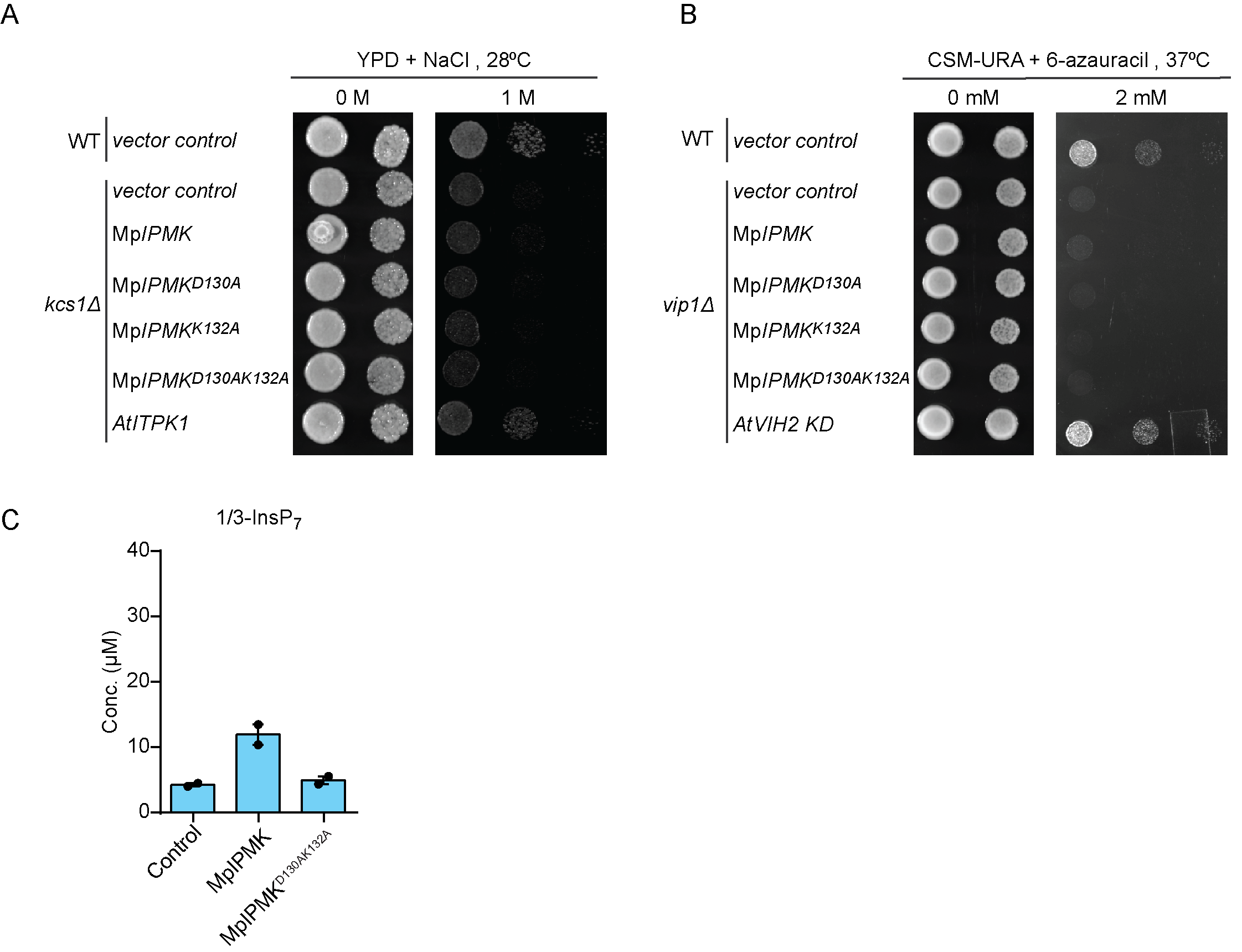

Supplement: S8 Fig — A. Complementation of the yeast kcs1∆-associated growth defects by the ectopic expression of MpIPMK. Wild-type and kcs1∆ yeast transformants (BY4741 background) carrying designated plasmids were spotted in 8-fold serial dilution onto YPD with and without NaCl incubated at 28ºC and 37⁰C. AtITPK1 served as positive control [30] and empty vector served as negative control. B. Complementation of vip1∆ -associated growth defects in yeast by ectopic expression of MpIPMK. The vip1∆ yeast strain transformed with the episomal pCA45 (URA3) plasmids carrying MpIPMK and kinase dead mutants were spotted in 8-fold serial dilutions onto uracil-free minimal medium in presence and absence of 6-azauracil. No rescue of phenotype was observed. AtVIH2 KD served as positive control [35] and empty vector served as negative control. C. Quantification of the reaction product of MpIPMK analyzed by CE-MS. Data represent means ± SEM (n = 2). (TIF) [file pgen.1011838.s008.tif]

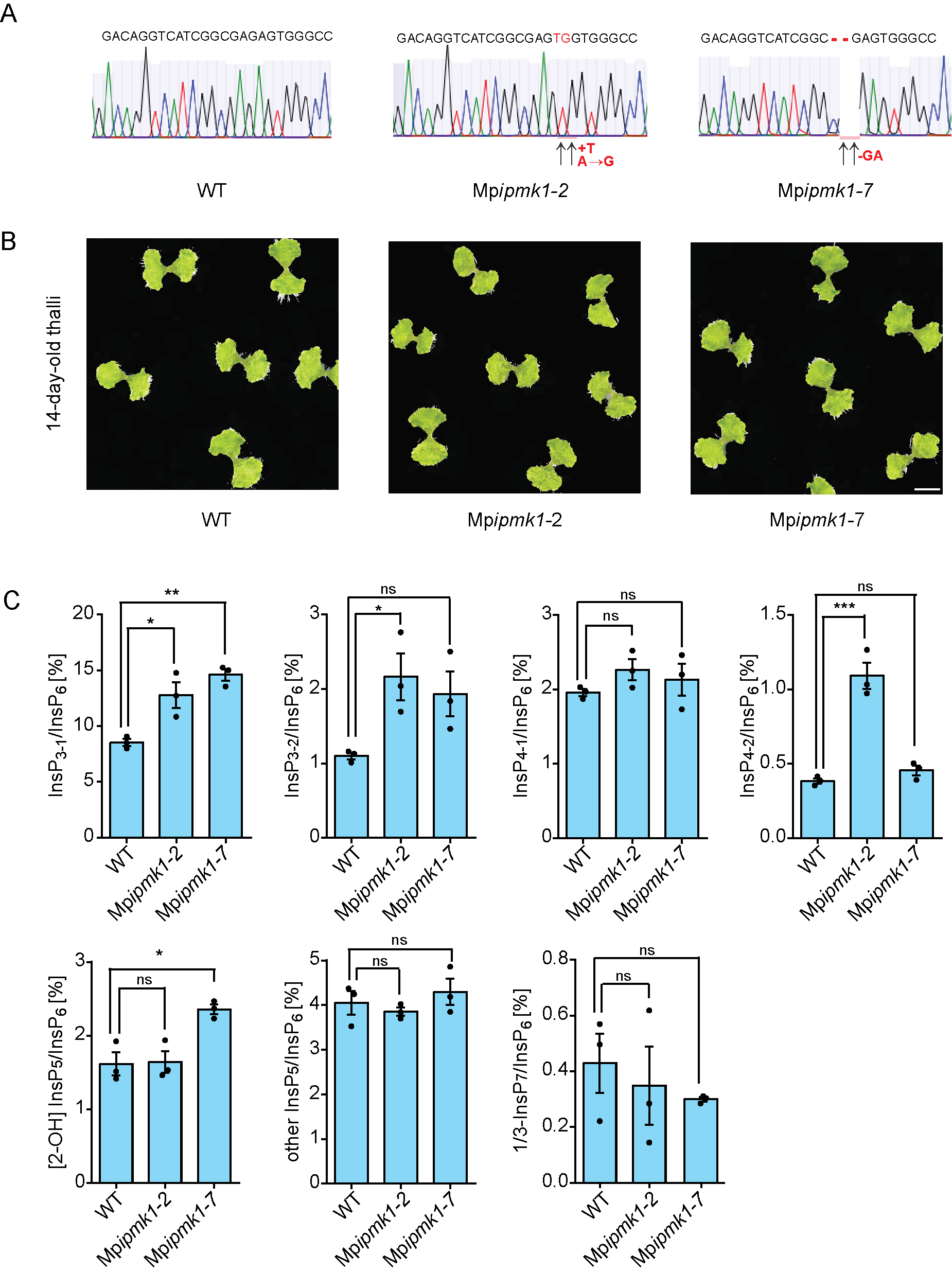

Supplement: S9 Fig — A. Chromatogram showing CRISPR/Cas9-edited nucleotide sequences of Mpipmk1.2 and Mpipmk1.7 compared with those of wild-type plants using chromatogram obtained from sequencing results. B. Photograph of 14-day-old thalli of wild-type, Mpipmk1.2 and Mpipmk1.7 plants. C. CE-MS analyses of different inositol phosphates isomers in wild-type and Mpipmk knockout plants. The InsP5 and InsP7 species were assigned by mass spectrometry and identical migration time compared with their relative standards. Two InsP4 and two InsP3 isomers were detected. Data are means ± SEM (n = 3, biological replicates). Inositol phosphates are represented as percentage to InsP6. Significant difference is determined by one-way ANOVA followed by Dunnett’s test (*P < 0.05, **P = 0.002 ***P < 0.001). (TIF) [file pgen.1011838.s009.tif]

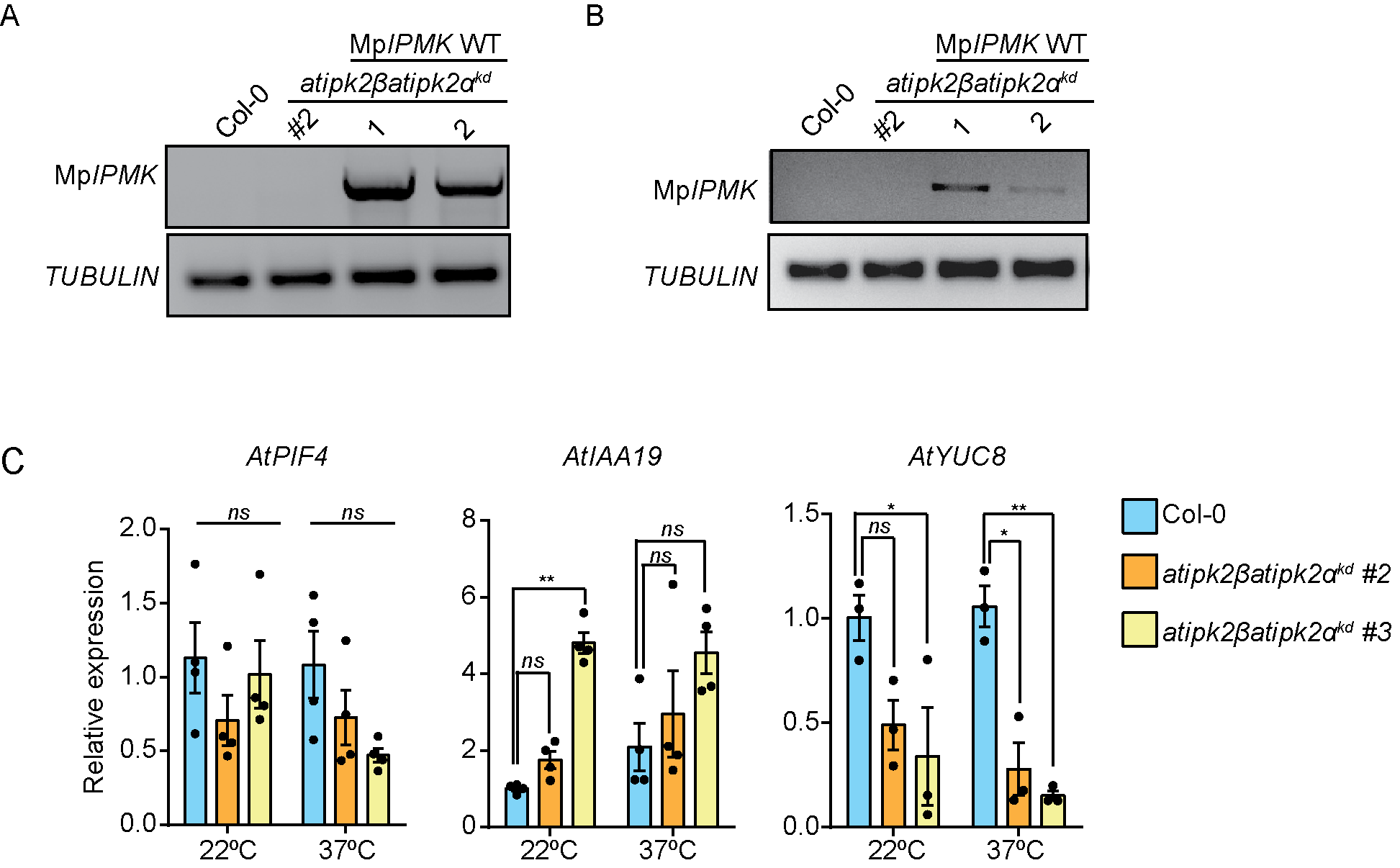

Supplement: S10 Fig — A. Genotyping PCR of atipk2βatipk2αkd lines expressing MpIPMK. The primers used for genotyping are mentioned in S1 Table. TUBULIN served as a reference gene. B. RT-PCR of atipk2βatipk2αkd lines expressing MpIPMK. The primers used for RT-PCR are mentioned in S1 Table. TUBULIN served as a reference gene. C. Quantitative RT-PCR (qRT-PCR) analysis of different genes involved in thermomorphogenesis between Col-0 and the atipk2βatipk2αkd lines after heat shock. 14-day-old seedlings were exposed to 37⁰C for 3 h and were harvested for qRT-PCR analysis. Transcript levels of the benchmark genes are presented relative PP2AA3 transcript. Values are means ± SEM (n ≥ 3, biological replicates). Statistical significance is determined by two-way ANOVA followed by Tukey’s test (*P < 0.05, **P < 0.001). (TIF) [file pgen.1011838.s010.tif]

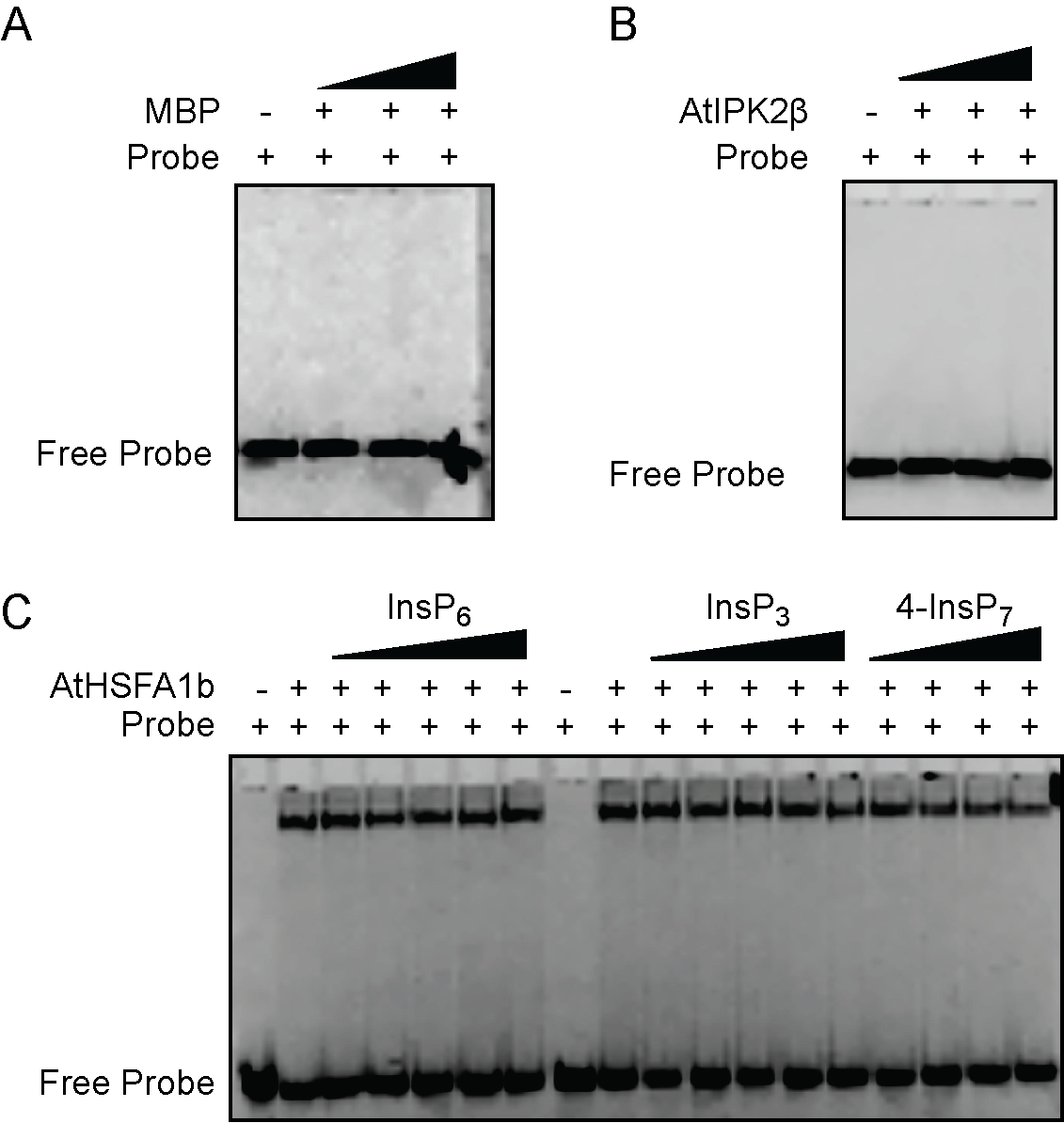

Supplement: S11 Fig — A and B. EMSA showing MBP, MBP-AtIPK2β do not bind directly to HSE element. 250 nM of the probe was used. MBP and MBP-AtIPK2β were used in the concentration ranging from 50- 200 nM. C. Ins(1,4,5)P3, InsP6 and 4/6-InsP7 don’t influence DNA-binding activity of heat shock transcription factor. 100 nM of HSF was pre-incubated with InsP6, InsP3 and InsP7 (10 nM, 50 nM, 100 nM and 10 µM) for 30 mins followed by incubation with FAM-labelled probe for 15 mins on ice. The complexes were resolved using 6% of native PAGE. (TIF) [file pgen.1011838.s011.tif]

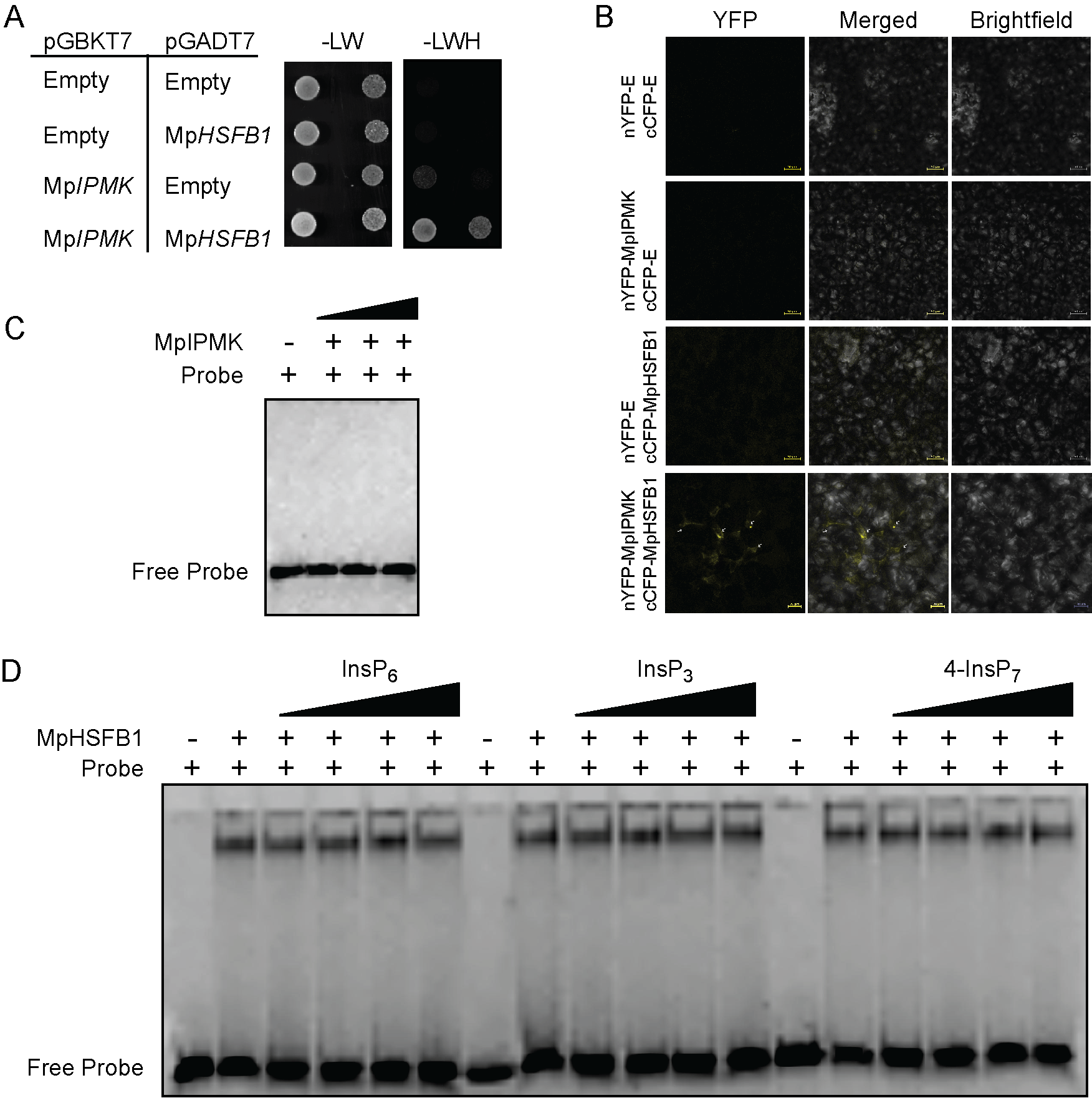

Supplement: S12 Fig — A. MpIPMK shows physical interaction with MpHSFB1 in vivo. AH109 yeast strain carrying the pGADT7-MpHSF and pGBKT7-MpIPMK plasmids were spotted on selective media. B. Transiently expressed MpIPMK interacts with MpHSFB1 in the nucleus of N. benthamiana cells. Different combination of co-expressed nVENUS and cCFP constructs were infiltrated in N. benthamiana. YFP represents the images taken with YFP filter and merge represents the overlay of YFP and brightfiled. Scale bar = 50 µm. C and D. EMSA showing MBP-MpIPMK do not bind directly to HSE element and PP-InsPs do not affect MpHSFB1 binding to HSE element. 250 nM of the probe was used. MpIPMK was used in the concentration ranging from 50- 200 nM. Ins(1,4,5)P3, InsP6 and 4/6-InsP7 don’t influence DNA-binding activity of heat shock transcription factor. 100 nM of HSFB1 was pre-incubated with InsP6, InsP3 and InsP7 (10 nM, 50 nM, 100 nM and 10 µM) for 30 mins followed by incubation with FAM-labelled probe for 15 mins on ice. The complexes were resolved using 6% of native PAGE. (TIF) [file pgen.1011838.s012.tif]
