# Supplementary material for: Conservation of heat stress acclimation by the IPK2-type kinases that control the synthesis of the inositol pyrophosphate 4/6-InsP7 in land plants
Source: PLoS Genet. 2025 Sep 11;21(9):e1011838. doi: 10.1371/journal.pgen.1011838 (PMC12445474; doi:10.1371/journal.pgen.1011838)
Supplement: Attachment — Submitted filename: Response to Reviewers.pdf [file pgen.1011838.s021.pdf]

Dear Dr. Laha,

I apologize for the delay in reviewing this manuscript, as we have difficulty securing reviewers. As you may see from the review comments, both reviewers find the identification of IPK2s as inositol pyrophosphate synthesizing enzymes and their involvement in heat stress very interesting. I think that it is a new finding that advances the function of inositol pyrophosphate 4/6-InsP7 in plants. However, the reviewers also raised serious questions on data interpretation, experiment design (proper controls used), and the sufficiency of evidence for the conclusion. I agree with the reviewer that necessary controls should be used in the ITC experiments and the EMSA assays in order to draw a convincing conclusion. The IP3/4/5 kinase activities in this manuscript should be compared with these previously reported. In addition, how AtIPK2α enhances the DNA-binding and transcription activity of AtHSFA1b should be addressed. In light of these concerns, I recommend a major revision. I would encourage a resubmission after the questions and concerns are addressed.

Dear Dr. Tan, dear Editorial Team,

We would like to express our sincere thanks to the editor and both the reviewers for their thorough evaluation of our manuscript and for providing constructive feedback. We have addressed all of their concerns primarily by conducting additional new experiments and revising the text as recommended, which have significantly improved the manuscript. Our responses are highlighted in blue. Please find below the key findings that we have incorporated in the revised manuscript:

i) As indicated by both the referee, we have analysed PP-InsP species from members of Pteridophyta group to further strengthen our conclusion that 4/6-$InsP_7$ is the major form of $InsP_7$ isomers in the studied land plant tissue extracts, please see revised Fig. 1A and Supplementary Fig. 1.

ii) As instructed by the referee 2, we have performed kinetic analyses using known canonical substrates of IPK2 and found that InsP3 is preferred substrate *in vitro* (new Supplementary Fig. 2I). Notably, the Km for ATP for $InsP_6$ of AtIPK2 is similar to canonical mammalian IP6K and Arabidopsis ITPK1, suggesting that $InsP_6$ is likely a physiological substrate of AtIPK2. We have discussed these points in the revised manuscript.

iii) Following the Reviewer 1 suggestion, we have generated AtIPK2α overexpression lines and found that AtIPK2 OE lines show augmented shoot thermomorphogenesis compared to its isogenic wild-type plants (new Fig. 4K and 4L).

iv) We have performed additional control ITC experiments (new Fig. 1C, Supplementary Fig. 2D and 2E) and have repeated all the EMSA experiments (revised Fig. 9) following the instruction of reviewer 2.

v) As instructed by both the referee, we show that Arabidopsis IPK2/Marchantia IPMK physically interacts with HSF using Y2H and BiFC assays (new Fig. 9A and 9B; Supplementary Fig. 12B). We investigated the consequences of these physical interaction and found that IPK2 potentiates the DNA-binding activity of HSFs (new Fig. 9C-L). These data provide insights into the IPK2-controlled heat stress acclimation through modulating HSF activity.

We hope that, with these substantial amendments, the manuscript now meets the standards of *Plos Genetics*.

Reviewer #1: The authors conducted CE-MS analysis and identified that 4/6-InsP7 is the predominant PP-InsP species present in Arabidopsis and liverworts. They also identified the enzymes responsible for the biosynthesis of 4/6-InsP7 and further tested the evolutionary conservation of these enzymes in generating 4/6-InsP7 and regulating heat stress tolerance. The findings are quite interesting, and I have a few comments.

We are grateful to the reviewer for the encouraging remarks.

1. Line 118: The authors state, "To investigate whether 4/6-InsP_7 is ubiquitous across land plants." However, in the manuscript, they only examine the inositol phosphate profiles from liverworts and Arabidopsis, which seems insufficient to support the claim of "land plants." It would be better to include several land plants of evolutionary significance or cite relevant literature to substantiate this claim.

We completely agree with the reviewer's suggestion. To further strengthen our findings, we repeated the CE-MS analyses, including two species from the Pteridophyta group (namely *Nephrolepis* sp. and *Dryopteris* sp.), along with representative members of the bryophytes and angiosperms. Our analyses show that 4/6-InsP_7 is the predominant PP-InsP isomer in all of these studied land plants. The corresponding data have been incorporated into the revised Figure 1A.

To be more accurate as pointed out by the referee, we have rephrased our statement as following "Altogether, these findings revealed that 4/6-InsP_7 is the predominant InsP_7 isomer detected in the specific tissue extracts of the embryophytes used in our study". Please see lines 134 to 136. We have also revised the respective subheading as "4/6-InsP_7 is ubiquitous the studied land plants"

2. If possible, it would be beneficial to generate an atipk2β(kd)atipk2α mutant to further support the conclusion that AtIPK2α and AtIPK2β act redundantly to regulate the synthesis of 4/6-InsP7 in Arabidopsis.

This is an important point made by the referee. We would like to emphasize that the development and validation of the *atipk2β$^{-/-}$atipk2α^{kd}* stable knockdown lines, along with their corresponding complementation lines, required nearly two years of dedicated effort. Similarly, the generation and validation of *atipk2β^{kd}atipk2α$^{-/-}$* plants and their complementary lines are expected to be equally time-intensive. Although the development of these lines is currently underway, it remains difficult at this stage to provide a definitive timeline for when experiments involving these lines can be conducted. To strengthen our conclusion, we presented findings in the first submission that single knockout of *atipk2α* and *atipk2β* do not exhibit any defects in 4/6-InsP_7 production (Fig. 3A). Consistently, we showed that the *atipk2β$^{-/-}$atipk2α^{kd}* plants exhibit defective production of 4/6-InsP_7 (Fig. 2D). Moreover, our findings reveal that the expression of AtIPK2β can restore the *atipk2β$^{-/-}$atipk2α^{kd}* plants-associated defects (Fig. 4H-J).

3. In Supplementary Data Set 3, which column represents the transcripts of IPK2 under heat stress? The authors need to label the treatments clearly.

Thank you for pointing this out. We have now clearly highlighted the column in the revised manuscript and prepared a graphical representation for further clarity, please see the revised Supplementary Data Set 3. To further strengthen the data, we have also performed qPCR analyses to study the expression levels of *AtIPK2α/β* after heat treatment. Our qPCR analyses show that heat stress augments the expression of AtIPK2*α/β* (new Supplementary Figure 4B).

4. Lines 342-355: The authors utilize the ipk2Δ knockout strain to test the functional conservation among yeast, *M. polymorpha, and Arabidopsis*. What is known about the role of IPK2 in yeast? The authors need to introduce the function of yeast IPK2 in the introduction.

Thank you for the suggestion. We have now discussed the role of yeast IPK2 in the Introduction section as instructed by the referee. Please see lines 64 to 69.

5. I am curious whether the overexpression (OE) of IPK2 could significantly enhance the heat tolerance of Arabidopsis plants. The manuscript only discusses the phenotypes of complementary lines and does not mention the OE lines.

We are grateful for this suggestion. We have generated independent Arabidopsis transgenic lines expressing *AtIPK2α* in translational fusion with a GFP tag under the control of a constitutive 35S promoter. We found that the independent overexpression lines exhibit increased hypocotyl length compared to its isogenic wild-type plants when exposed to heat stress (new Fig. 4K and 4L; Supplementary Fig. 5D). These findings further reinstate that AtIPK2 activity is critical for heat stress acclimation in Arabidopsis. The corresponding data have been incorporated as in the revised manuscript.

6. The authors performed EMSA assays and concluded that the AtIPK2α protein enhances the DNA-binding and transcription activity of AtHSFA1b. Is the IPK2 protein localized in the nucleus? Does AtIPK2α interact with AtHSFA1b at the protein level? BiFC, split-luciferase, or Co-IP experiments would be necessary to validate this interaction. The incubation of AtHSFA1b with Ins(1,4,5)P3, InsP6, and 4-InsP7 did not affect the DNA-binding activity of AtHSFA1b. Therefore, AtIPK2 appears to have biological functions in catalyzing the formation of 4/6-InsP7 and enhancing the DNA-binding and transcription activity of AtHSFA1b. Do these two functions occur in the same subcellular location, such as the nucleus? Is AtIPK2 localized in both the cytosol and the nucleus? The authors need to address these questions.

These are important points raised by the referee. Previous studies by Xu et al., 2005 *Plant physiology* (PMID: 15618435) and Xie et al., 2003 *The Plant Cell* (PMID: 12566584) already reported that AtIPK2*α and* AtIPK2β localize in the nucleus, respectively. Consistent with these previous findings, our data indicate that AtIPK2 localizes in nucleus when ectopically expressed in *N. benthamiana*. Please see below Response Figure 1.

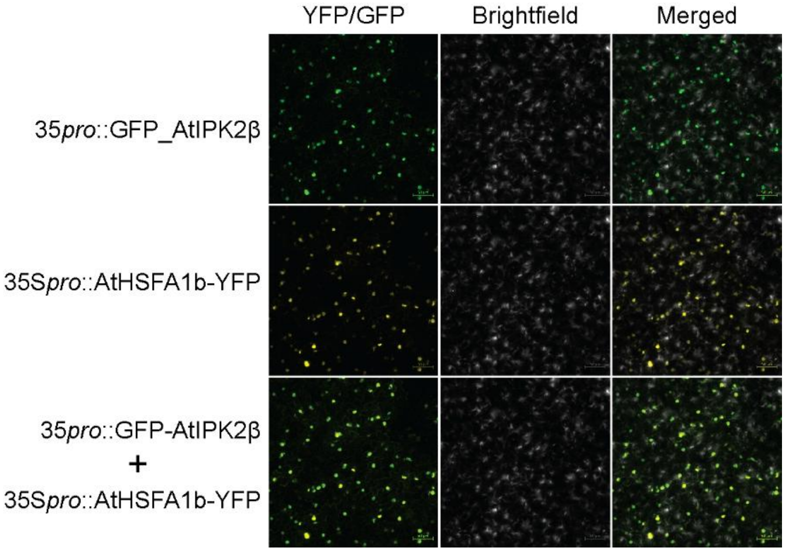

Response Figure 1: Co-localization of AtHSFA1b and AtIPK2β in *N. bethamiana* leaves. AtHSFA1b-YFP and AtIPK2β-GFP were co-infiltrated in *N. bethamiana* leaves. Confocal microscopy showing co-localization of AtPK2β and AtHSAF1b in *N. benthamiana* leaves.

Our co-localization experiment suggests that AtIPK2s with AtHSFA1b in *N. benthamiana* and found that both the proteins co-localize in nucleus (Response Fig. 1).

We would like to point out that although InsP and PP-InsP did not influence the DNA-binding activity of AtHSFA1b *in vitro*, we found that the catalytic activity of AtIPK2 is critical for augmenting transcriptional activity of AtHSFA1b *in planta* (Fig. 8B). This finding highlights that AtIPK2-derived (PP)-InsP controls the AtHSFA1b activity *in planta*. Our EMSA assays revealed that IPK2 can potentiates the DNA-binding activity of HSF in catalytic-independent manner (revised Fig. 9). At present, the factors governing the catalytic-dependent and catalytic-independent functions of IPK2 in regulating HSF transcriptional activity remain unclear. We hope to address this interesting point in future studies. We have discussed this point in the discussion part, please see line (580- 591).

As suggested by the referee, we show that indeed AtIPK2α interacts physically with AtHSFA1b using Y2H and BiFC assays (new Fig. 9A and 9B). These new findings are included in the revised manuscript. Furthermore, we show that similar to Arabidopsis IPK2, Marchantia IPMK also physically interacts with MpHSFB1 (new Supplementary Fig. 12B).

Reviewer #2: The manuscript by Yadav and colleagues describes the identification of IPK2s from the model plants Arabidopsis and Marchantia as inositol pyrophosphate synthesizing enzymes involved in heat stress adaptation. The manuscript reports a number of interesting findings including new mutant alleles for IPK2s and a strong genetic connection between IPK2 function and plant heat stress responses. However, it is unclear of indeed InsP6 is the best substrate for IPK2s and if the enzyme directly regulates cellular 4/6-InsP7 levels as well as the transcriptional activity of plant heat shock transcription factors. I summarize my comments and suggestions below:

We are thankful to the reviewer for the constructive feedback. Please find below our detailed response that addresses the important concerns raised by the referee.

1. MINOR Introduction, Line 77-78. There is, to the best of my knowledge, no strong evidence that plant PPIP5Ks are $InsP_6$ kinases. A Mpvip1ge mutant in Marchantia polymorpha has wild-type like levels of 1-$InsP_7$, increased levels of 5-$InsP_7$ and decreased levels of 1,5-$InsP_8$ (https://pubmed.ncbi.nlm.nih.gov/39531477/). This suggests that at least in planta, Vip1 is not an InsP6 kinase, but may use exclusively use 5-$InsP_7$ as substrate to generate 1,5-InsP8.

We agree with the reviewer that there is no strong evidence from existing literatures supporting plant PPIP5Ks function as an $InsP_6$ kinase *in planta*. Notably, Riemer et al (PMID: 38834073) reported that 1/3-$InsP_7$ level is completely affected in the *vih2-4* mutant plants under phosphate resupply indicating that VIH2 function is critical for maintaining 1/3-$InsP_7$ level under this specific condition. In contrast, *vih2* lines did not exhibit changes in 1/3-$InsP_7$ level under control condition (Fig. 4C, right-middle panel by Riemer et al., 2021). A study by Zhu et al., reported that AtVIH2KD catalyzes the synthesis of 1/3-$InsP_7$ from $InsP_6$ and $InsP_8$ from 5-$InsP_7$ *in vitro* (Zhu et al, PMID: 31436531; Figure 5A, Figure 5-Figure supplement 2).

However, as noted by the reviewer, Mp*vip1*^{ge} mutant in *Marchantia polymorpha* has wild-type like levels of 1-$InsP_7$, increased levels of 5-$InsP_7$ and decreased levels of 1,5-$InsP_8$ (https://pubmed.ncbi.nlm.nih.gov/39531477/). Given that the lack of strong genetic and biochemical evidence for the $InsP_6$ kinase activity of plant VIH as correctly pointed out by the referee, we have now rephrased the sentence in our revised manuscript, please see lines 82 to 84: "*Arabidopsis* Vip1 isoforms, VIH1 and VIH2 possess 1-kinase activity, produce 1-$InsP_7$ and 1,5-insP_8 *in vitro* and contribute to $InsP_8$ synthesis *in planta*."

2. MINOR Line 92-94. 4/6-InsP7 levels have been reported for Arabidopsis seedling and for Marchantia Tak-1 plants here as well https://pubmed.ncbi.nlm.nih.gov/39531477/.

Thank you for pointing this out. We have cited the recent study by Laurent et al., Plos Genetics 2024 that have reported 4/6-$InsP_7$ in plants, as suggested by the referee. Furthermore, we also incorporated the following citations that also reported 4/6-$InsP_7$ in Marchantia Tak-1 plant extracts:
Pullagurla et al., 2024 (https://pubmed.ncbi.nlm.nih.gov/39190827/) and
Chalak et al., 2024 (https://pubmed.ncbi.nlm.nih.gov/39404446/).

3. MAJOR Line 126-127. 4/6-InsP7 levels have been previously determined in Arabidopsis (references cited in the introduction + https://pubmed.ncbi.nlm.nih.gov/39531477/, see above) seedlings and in Marchantia polymorpha Tak-1 and various mutants in the PP-InsP biosynthesis and catabolic pathway (https://pubmed.ncbi.nlm.nih.gov/39531477/). In this previous study, both in Arabidopsis and in Tak-1 4/6-InsP7 levels are not significantly higher compared to either 1-InsP7, 5-InsP7 or 1,5-InsP8 (compare Fig. 1F and 3G in https://pubmed.ncbi.nlm.nih.gov/39531477/, respectively). Given that the absolute abundance of a certain PP-InsP species is poorly correlated with its biological signaling capacity, I would consider removing the statement that 4,6-InsP7 is "the most abundant InsP7 isomer". It would also be helpful if the authors could plot the total concentrations of InsPs and PP-InsPs per g of tissue weight, rather than as a fraction of InsP6 levels.

This is an important observation made by the referee! We would like to emphasize that four peer-reviewed publications to date report the presence of 4/6-$InsP_7$ in Arabidopsis and Marchantia extracts:

i) Riemer et al., Molecular Plant, 2021 (https://pubmed.ncbi.nlm.nih.gov/34274522/): The authors observed elevated levels of 4/6-$InsP_7$ compared to 1/3- and 5-$InsP_7$ in Arabidopsis plants grown in **hydroponics**. Please see Figures 3C and 4C in Riemer et al.'s study.

ii) Pullagurla et al., Plant Physiology, 2024 (August): The authors found that the level of 4/6-$InsP_7$ was consistently higher than that of 1/3-$InsP_7$ and 5-$InsP_7$ in **two-week-old** *Marchantia polymorpha* extracts. Please see Figures 4C, 5C, and 1E in Pullagurla et al.'s study (https://pubmed.ncbi.nlm.nih.gov/39190827/).

iii) Chalak et al., Biochemistry, 2024 (October): As shown in Figure 2D of this study, the level of 4/6-$InsP_7$ was significantly higher than that of other $InsP_7$ isomers in **two-week-old** *Marchantia polymorpha* extracts (https://pubmed.ncbi.nlm.nih.gov/39404446/).

iv) Laurent et al., PLOS Genetics, 2024 (November): As noted by the reviewer, the levels of 4/6-$InsP_7$ were similar to those of 5- and 1/3-$InsP_7$ **in three-week-old** *Marchantia polymorpha* extracts and **two-week-old** whole seedlings (https://pubmed.ncbi.nlm.nih.gov/39531477/).

Notably, all PP-InsP analyses in these above-mentioned studies were conducted using the same CE-MS setup at the Henning Jessen's lab, University of Freiburg, Germany. The differences across these studies can be attributed to variations in plant age (two-week vs. three-week), tissue type (whole seedlings vs. shoot-only), and growth conditions (plate vs. hydroponics). These factors may explain the differing results observed by the various groups. We have incorporated all these citations in the revised manuscript.

In any case, we completely agree with the reviewer's statement that "the absolute abundance of a certain PP-InsP species is poorly correlated with its biological signaling capacity." In response to the reviewer's suggestion, we have repeated the whole experiment, now including new plant species from the Pteridophyte lineage, and presented the data as **pmol/mg**. We again observed that 4/6-$InsP_7$ is the predominant PP-InsP isomer in the specific plant tissue

extracts analysed (Fig. 1A). We have revised our statement to reflect this: “Altogether, these findings revealed that 4/6-$InsP_7$ is the predominant $InsP_7$ isomer detected in the specific tissue extracts of the embryophytes used in our study.” We have also revised the respective subheading as “4/6-$InsP_7$ is ubiquitous in the studied land plants”. Please see line 123 to 137.

5. MINOR A structural superposition comparing HsIP6K3 and AtIPK2 in Ca representation should be presented, with corresponding structural alignment statistics (root mean square deviation in Angstroems comparing X corresponding Calpha atoms matching between the two structures) in revised Figure S6C.

We are grateful for the detailed instruction. We have performed the structural superposition comparing HsIP6K3 and AtIPK2 and provided the root mean square deviation in Figure legends in Supplementary Fig. 2A as instructed by the referee. The corresponding data has been included in the revised manuscript.

6. MAJOR: Lines 168-180 and Figure 1C. The ITC experiments should to be repeated with appropriate controls. It is not sufficient to report the raw heats from injections of substrate (in the syringe) into the enzyme (in the cell). Essential control experiments are 1) injection of substrate (in the syringe) into buffer (in the cell, 50 mM Tris Cl pH 7.0 and 0.5 mM EDTA) and 2) injection of buffer (in the syringe, 50 mM Tris Cl pH 7.0 and 0.5 mM EDTA) into enzyme (in the cell). The raw heats of these control experiments should be plotted alongside the actual experiment in the upper panels of Figure 1C. Titration of InsPs into free buffer is usually associated with large DPs, this needs to be checked and corrected for. Binding stoichiometries (N) should not be fixed during data fitting and should be reported alongside the derived dissociation constants. It is presently hard to understand how, for example, the raw heats in the top left experiment in panel 1C could be fitted at to the binding curve. The method section should include the injection volumes for each injection and the method of fitting. How was the pH of the ligand in ITC buffer dilutions adjusted? Why is the Kd derived by ITC (0.5 micromolar) drastically different from the Km estimate (~250 micromolar)?

We are grateful for the valuable suggestions provided. Accordingly, we have repeated the ITC experiments with the appropriate controls, 1) including injection of ligand (via syringe) and injection of buffer into the cell (new Fig. 1C) and 2) included a control where buffer was injected into the enzyme (new Supplementary Fig. 2D and 2E). We would like to emphasize that the injection of ligand (either $InsP_3$ or $InsP_6$) in buffer, in our experimental set up, alone does not result in a significant differential power (DP) signal, with values lower than -0.05 µcal/s on the y-axis scale (new Fig 1C). In contrast, the injection of ligand into enzyme produces a much larger DP signal, more than -0.1 µcal/s (revised Fig. 1D and 1E), demonstrating specific binding interaction rather than non-specific heat changes. Additionally, we have included the binding stoichiometry in the revised Fig. 1D and 1E. We did not fix the stoichiometric parameter (N) during data fitting and used a one-site binding model for the analysis. We apologize for the error in the previously reported pH of the ITC buffer; the correct pH is 8.0. We have now included details of injection volumes and data fitting methods in the Methods section. Moreover, the pH of the ligands after dilution in ITC buffer did not show any significant changes that could affect the results.

The reviewer correctly pointed out that there is a drastic difference between Kd (0.5 µM) and Km (approx. 1.8 mM) (Fig. 1D, 1E and Supplementary Figure 2I). Km value that represents the substrate concentration at which the enzyme's reaction rate is half of its maximum velocity, is different from Kd which points out how tightly a ligand bind to its receptor. Km was determined by monitoring the IPK2-derived product over the time whereas K_D was determined just incubating the ligand and protein without any ATP.

We agree with the reviewer that drawing conclusions about substrate specificity based solely on K_D values may not be appropriate. Given its higher negative charge, $InsP_6$ is likely to bind more tightly to AtIPK2 than $InsP_3$, which is consistent with our experimental observations (Fig. 1D and 1E). Accordingly, we have moderated our interpretation of the K_D data and revised the text to reflect this. Please see the updated text in lines 192 to 200.

7. MAJOR The newly reported kinase activity for IPK2a needs to be compared to the well established IP3/4/5 kinase activities previously reported for this enzyme. AtIPK2a has been previously characterized as an InsP3 kinase, which can also accept InsP4 and InsP5 as potential substrates (https://pubmed.ncbi.nlm.nih.gov/12226109/) Specifically, AtIPK2a has been characterized to phosphorylate 1,4,5-InsP3 to 1,4,5,6-InsP6, or 1,3,4,5-InsP4 to 1,3,4,5,6-InsP5 i.e. phosphorylating the 6-position of the inositol ring, with Km's in the low micromolar range, and with Vmax of ~ 100 nmol min-1 mg-1. In contrast, the Km for InsP6 is 250 micromolar, with a Vmax of only 2 nmol min-1 mg. This suggests that InsP6 is a very poor substrate for AtIPK2, when compared to InsP3 or InsP4 (https://pubmed.ncbi.nlm.nih.gov/12226109/). The authors should consider performing comparative enzyme kinetics with AtIPK2a and InsP3, InsP4 and InsP6 as substrate to define the best in vitro substrate for this enzyme.

Following the Reviewer's suggestion, we have conducted kinetic analysis of AtIPK2 proteins using its known well-established *in vitro* substrate, Ins(1,4,5)P_3 and compared with $InsP_6$. In line with published results (Stevenson-Paulik et al., JBC 2002, PMID: 12226109), we observed that the velocity of Ins(1,4,5)P_3 kinase activity of AtIPK2 is much higher than that of $InsP_6$ kinase reaction. The Km for ATP for Ins(1,4,5)P_3 of AtIPK2 is in micromolar range which is in consistent with the published data (new Supplementary Fig. 2I). These findings suggest that $InsP_3$ is indeed the preferred substrate *in vitro* as pointed out by the referee. Please note that we could not include the $InsP_4$ isomer in our analysis, as the suppliers were unable to deliver the isomer due to ongoing issues with material shipment and import. Nonetheless, we suspect the reaction velocity would be consistent with previously published findings as pointed out by the referee (Stevenson-Paulik et al., JBC 2002, PMID: 12226109).

We would like to point out here that Km for ATP for $InsP_6$ for AtIPK2 proteins are between 1.8 to 2 mM (new Supplementary Fig. 2I). Notably, these Km values are comparable with mammalian IP6K (Voglmaier et al., PNAS 1996: 8633060) and Arabidopsis ITPK1 (Riemer et al., Mol Plant 2021, PMID: 34274522; Whitfield et al., Biochem J, PMID: 32706850). Given that kinetic parameters associated with $InsP_6$ kinase activity of AtIPK2 resemble with Arabidopsis ITPK1, it is likely that $InsP_6$ is a physiological substrate of AtIPK2. We have incorporated these new findings (please see lines 192 to 200) and revised our discussion highlighting these important points (lines 528 to 533).

We agree with the reviewer that the velocity of the $InsP_6$ kinase reaction catalyzed by AtIPK2s is unexpectedly slow compared to its well-known canonical substrates, such as Ins(1,4,5)P_3 isomer. This point was already acknowledged in our initial manuscript (line 198 to 199 in the revised manuscript). It is also worth noting that, similar to AtIPK2, Arabidopsis ITPK1, which converts $InsP_6$ to 5-$InsP_7$ *in vitro*, *in vivo*, and *in planta*, exhibits a drastic slower $InsP_6$ kinase reaction velocity compared to its previously reported canonical substrate, Ins(3,4,5,6)P_4. For example, Whitfield et al. reported that the reaction velocity of ITPK1 with its canonical *in vitro* substrate, Ins(3,4,5,6)P_4, is 8640 nmol/min/mg (Whitfield et al., Biochem J 2020 PMID: 32706850), whereas its velocity with $InsP_6$ ranges from 17 to 40 nmol/min/mg (Whitfield et al., Biochem J 2020 PMID: 32706850; Riemer et al., Mol Plant 2021 PMID: 34274522). Interestingly, Arabidopsis *itpk1* knockout lines show altered levels of $InsP_4$ and $InsP_5$ isomers, but $InsP_6$ levels remain unaffected (Riemer et al., Mol Plant 2021 PMID: 34274522; Parvin-Laha et al., Plant Phys PMID: 36124979; Kuo et al., Plant J 2018 PMID: 29779236).

Remarkably, these *atitpk1* mutant lines are defective in 5-InsP$_7$ production (Riemer et al., Mol Plant 2021 PMID: 34274522; Parvin-Laha et al., Plant Phys PMID: 36124979), highlighting that InsP$_6$ kinase activity of AtITPK1 is compromised. These observations reinstate that ITPK1 recognizes independently both InsP$_4$ and InsP$_6$ as substrates. While the metabolic fate of InsP$_4$ phosphorylated product is yet to be elucidated, ITPK1 converts InsP$_6$ to 5-InsP$_7$ *in planta*. Consistently, we reported that the overexpression of MpITPK1 in *M. polymorpha* leads to the accumulation of 5-InsP$_7$ reinforcing that ITPK1 functions as InsP$_6$ kinase *in planta* (Pullagurla et al., Plant Phys 2024, PMID: 39190827). These findings also highlight that *in vitro* kinetic data may not always reflect *in vivo* enzymatic activity.

Our CE-MS analyses show that IPK2-deficient seedlings are defective in InsP$_{4-5}$ metabolism but does not exhibit changes in InsP$_6$, the precursor of InsP$_7$ (Fig. 2D). If alterations in InsP4/5 species were responsible for the decreased levels of InsP$_6$, it would be expected that all InsP$_7$ isomers would be affected in the IPK2-deficient plants. However, IPK2-deficient plants exhibit a specific reduction in 4/6-InsP$_7$ production, while other InsP$_7$ isomers remain unaffected (Fig. 2D). These findings emphasize that similar to AtITPK1, AtIPK2 phosphorylates different InsP substrates independently including InsP$_6$ to produce 4/6-InsP$_7$ *in planta*.

Based on the current data the statement line 216-219 is not supported by robust experimentation.

In the revised version of our manuscript (lines 224 to 226), we stated, “Collectively, our analyses identified a previously unreported non-canonical function of Arabidopsis IPK2 proteins as PP-InsP synthase, catalyzing the phosphorylation of InsP$_6$ to generate 4/6-InsP$_7$ *in vitro*.” We hope the reviewer agrees that this statement does not imply that InsP$_6$ is the preferred substrate over InsP$_3$, either *in vitro* or *in planta*. Additionally, we show that when incubated with InsP$_6$, IPK2 primarily produces 4/6-InsP$_7$ as the major InsP$_7$ isomer *in vitro* (Fig.1H, 1I and Supplementary Fig. 2J-L).

In conclusion, we agree with the reviewer that InsP$_6$ is not the preferred substrate *in vitro* compared to the well-known canonical substrates of AtIPK2, InsP$_3$ and InsP$_4$. We have performed new experiment to estimate Km for ATP for InsP$_3$ substrate and included the dataset (new Supplementary Fig. 2I). Accordingly, we have revised the manuscript and toned down completely. The point we would like to emphasize here that our findings suggest that AtIPK2 phosphorylates both InsP$_{4/5}$ and InsP$_6$ independently *in planta*. The metabolic fate of the product derived from IPK2 reaction using InsP$_{4/5}$ as substrates is not clear at the moment as InsP$_6$ level remains unaffected. Specific reduction in 4/6-InsP$_7$ but not in 5-, and 1/3-InsP$_7$ isomers despite no changes in InsP$_6$ in the IPK2-deficient plants reinforce that AtIPK2 contributes to the synthesis of 4/6-InsP$_7$ *in planta*.

8. MAJOR: Figure 2C The atipk2b/atipk2a knock-down line contains higher levels of InsP3, InsP4 and InsP5 but only very small variations in 4/6-InsP7. How come the difference in for example InsP5 levels between Col-0 (~1.6 pmol mg-1 FW) and atipk2b/atipk2akd line2 (~3 pmol mg-1 FW) are labeled statistical insignificant, while in the case of 4/6-InsP7 much smaller differences (Col-0 ~0.6 pmol mg-1 FW vs. 0.4 pmol mg-1 FW) are labeled as highly significant? Why are the total levels of InsPs/PP-InsPs so different for the Col-0 control when comparing the experiments shown in Figure 2A and 2D)? The authors should provide further details on the statistical tests used and discuss the possibility that the altered InsP3/4/5 levels may indirectly affect 4/5-InsP7 concentrations rather than the IPK2 enzymes themselves (see above, point #7).

This is an interesting point. As noted in the figure legend, we used a one-way ANOVA followed by Dunnett’s test to assess statistical significance. Our analysis showed no significant

differences in $InsP_5$ levels among the genotypes, as highlighted in Figure 2D. Please note that the error bars represent the standard error of the mean (SEM), not standard deviation (SD). Full details of the statistical analyses are provided in Supplemental Data Set 7 (Sheet 2 for Fig. 2D). While the differences are not statistically significant, it is evident that AtIPK2-deficient lines tend to show elevated $InsP_5$ levels as pointed out by the referee. We have also uploaded the raw data related to this Figure. We have revised the manuscript accordingly.

Regarding the other points highlighted by the referee that changes in InsP3/4/5 in the IPK2-deficient lines may indirectly affect 4/6-$InsP_7$. As discussed above, if the accumulated $InsP_{3/4/5}$ isomers in the IPK2-deficient plants contribute to $InsP_6$ production, then one would expect that $InsP_6$ levels would be affected in the *atipk2βatipk2α*^{kd} lines. Notably, we did not observe changes in $InsP_6$ level between wild-type and the *atipk2βatipk2α*^{kd} seedlings emphasizing that compromised levels of $InsP_{3/4/5}$ in the IPK2-deficient lines does not affect $InsP_6$ production in seedlings (Fig. 2D and 2E). This is not completely surprising as previous studies have reported that despite the drastic changes in $InsP_{4/5}$ levels in the *atitpk1* knockout lines, $InsP_6$ levels remain unaffected in the ITPK1-deficient lines (Riemer et al., Mol Plant 2021 PMID: 34274522; Parvin-Laha et al., Plant Phys PMID: 36124979; Wang et al., Plant Phys 2021 PMID: 35235674- Fig. 3B). However, 5-$InsP_7$ level is significantly reduced in the *atitpk1* knockout plants compared to the wild-type seedlings.

Given that $InsP_6$ levels remain unchanged between wild-type and *atipk2β atipk2α*^{kd} lines, it is unlikely that altered levels of $InsP_{3/4/5}$ contribute to changes in 4/6-$InsP_7$ without affecting $InsP_6$. Since $InsP_6$ serves as the precursor for $InsP_7$ isomers, fluctuations in its levels would be expected to impact not only 4/6-$InsP_7$ but also other PP-InsP isomers. Consistent with a specific role for AtIPK2 in regulating 4/6-$InsP_7$, we observed a selective reduction in this isomer, while other $InsP_7$ species remained unaffected in the AtIPK2-deficient lines. We are unaware of any study that shows 5-$InsP_7$ or any other $InsP_7$ species can be produced in $InsP_6$-independent manner in plant.

One can envision scenarios where $InsP_3$ or $InsP_4$ is phosphorylated by IPK2/IPMK to produce 4/6-PP-$InsP_2$ or 4/6-PP-$InsP_3$. These PP-InsP isomers could then be phosphorylated by ITPK1 and IPK1 to make 4/6-$InsP_7$ without forming canonical $InsP_6$ precursor. However, such hypothetical scenario is not experimentally tested and beyond the scope of this study. Nevertheless, we have now discussed this possibility by including the following sentence in the discussion part "Future work awaits to clarify whether IPK2/IPMK contributes to 4/6-$InsP_7$ in an $InsP_6$-independent manner using InsP3/4/5 as substrates".

10. MAJOR 283-297 and Figure 3A. Consistent with Figure 2C, 4/6-InsP7 levels are reduced in Figure 3A when comparing the Col-0 wild type with the atipk2b/atipk2a knock-down lines. During heat stress, InsP4/6 levels are reduced in Col-0 and in the mutant lines, which to me would suggest that heat stress-induced changes of 4/6-InsP7 levels occur independent of atipk2a and atipk2b. Can the authors explain why (line 296-297) - "Collectively, our data suggests that AtIPK2α and AtIPK2β function together to control cellular levels of 4/6-InsP7 during heat stress in planta."

We are grateful for pointing this out. We agree that the reduction in 4/6-$InsP_7$ levels observed in Col-0 plants after 5 hours of heat stress may involve AtIPK2-independent mechanisms, potentially including the activation of specific phosphatases. We have corrected and revised our text accordingly.

To get further insights, we repeated the experiment with a 3-hour heat stress treatment. Notably, under these conditions, 4/6-$InsP_7$ levels in Col-0 plants remained comparable to those in the control. In contrast, a significant reduction in 4/6-$InsP_7$ was observed in the

AtIPK2-deficient lines after 3 hours of heat stress (Supplementary Fig. 4C). As of now, we do not understand the mechanism behind 4/6-$InsP_7$ reduction during heat stress in wild-type plants treated for 5h, this could be investigated in future studies.

11. MAJOR: Figure 5D, as outlined above it would be worthwhile to compare the InsP6 kinase activity of MpIPMK with its ability to phosphorylate InsP3 and InsP4.

We would like to highlight that, in contrast to AtIPK2, the purified MpIPMK protein retains activity for only up to 2-3 hours after purification, which presents a challenge for conducting reliable kinetic analyses. After testing multiple conditions, we identified a relatively improved PBS-based buffer system that enabled us to carry out a pilot experiment. Our kinetic studies with the purified MpIPMK protein indicated that, similar to Arabidopsis AtIPK2, Ins(1,4,5)P_3 is the preferred *in vitro* substrate compared to $InsP_6$. However, due to substantial variability in the Km values for both $InsP_3$ and $InsP_6$, likely resulting from limited enzyme stability and sub-optimal enzymatic activity, we decided not to include this dataset in the revised manuscript despite repeated attempts under our experimental conditions.

12. MAJOR: Figure 6B. Would the accumulation of another $InsP_3$ peak in the Mpipmk knock-out line not argue for this InsP3 being a preferred in vivo substrate for MpIPMK? In line with this, InsP3 levels are up in Supplementary Figure 8C, again indicating that the lower 4/6-InsP7 levels in this mutant could be caused by a missing InsP4 precursor required for 4/6-InsP7 biosynthesis.

We completely agree with the reviewer that *Mpipmk* mutant plants accumulate $InsP_3$ isomers, which is consistent with the published findings on canonical IPK2 activity towards $InsP_3$. As the referee noted, our CE-MS analyses also suggest that $InsP_3$ is likely a preferred *in vivo* substrate of MpIPMK. However, we did not observe any significant changes in the levels of $InsP_4$ or $InsP_5$ isomers in the MpIPMK-deficient lines. Additionally, $InsP_6$ levels remained unchanged between wild-type and *Mpipmk* mutant plants as demonstrated by independent methods such as PAGE, SAX-HPLC and CE-MS analyses (Fig. 6B and 6E). As discussed earlier, a reduction in $InsP_6$ would impair not only 4/6-$InsP_7$ production but also affect other $InsP_7$ isomers. Remarkably, we observed a specific reduction in 4/6-$InsP_7$, indicating that MpIPMK activity is crucial for maintaining cellular levels of 4/6-$InsP_7$. These findings highlight that changes in $InsP_3$ isomer and 4/6-$InsP_7$ level reflect independent substrate phosphorylation event by MpIPMK.

As discussed before and in response to the reviewer's suggestion, we have included the following sentence in the discussion: "Future work awaits to clarify whether IPK2 can produce 4/6-$InsP_7$ in an $InsP_6$-independent manner using InsP3/4/5 as substrates *in planta*."

13. MINOR: Figure 8A. Based on what criteria were the heat shock response target genes chosen for this analysis?

Thank you for your comment. The selection of heat shock proteins (HSPs) for our analysis was based on their well-established role in the heat shock response (HSR) in *Arabidopsis*. Heat shock proteins (HSPs), play a critical role in helping plants cope with elevated temperatures and the expression of these genes are regulated by Heat shock transcription factor (specifically, AtHSFA1s) (Yoshida et al., 2011 *Mol Genet Genomics* PMID: 21931939, Lie et al., 2011 *Plant Cell Environment* PMID: 21241330). In addition, auxin-related genes are also known to be upregulated under heat stress (Gray et al., 1998 *PNAS*, PMID: 9618562,

Sun et al., 2012. Plos genetics PMID: 22479194). We have now incorporated these citations in the revised manuscript.

14. MINOR: Figure 8B. Is it known that HSFA1b is regulating expression of HSP70, HSP22, HSP101, HSP17.6, HSP17.4, HSP18.1 and HSFA2 or how was this particular transcription factor selected? Also, there are many possible molecular scenarios that could explain why expression of AtIPK2a may lead to enhanced transcriptional activity of AtHSFA1b. What is the rational to focus on the direct interaction of the enzyme with the transcription factor, a rather unusual mode of transcription factor regulation? Have other scenarios been tested experimentally?

These are important points. The selection of heat shock proteins (HSPs) for our analysis was based on their well-established role in the heat shock response (HSR) in *Arabidopsis*. HSPs are key components of the plant's response to heat stress, and their expression is tightly regulated by heat shock factors (HSFs).

Arabidopsis contains 21 heat shock factors (HSFs) classified into three groups (A, B, and C), which are further divided into 14 subgroups (A1 to A9, B1 to B4, and C1) (Nover et al., 2001 *Cell Stress Chaperones* PMID: 11599559, Scharf et al., 2012 *Biochim Biophys Acta* PMID: 22033015). Among these, the A1 group includes four members: HsfA1a, HsfA1b, HsfA1d, and HsfA1e, which act as activators. The *hsfa1a hsfa1b hsfa1d* triple knockout mutant (*hsfa1a/b/d*) fails to induce the expression of most heat shock (HS)-inducible genes and exhibits significant defects in thermotolerance, indicating that HsfA1a, HsfA1b, and HsfA1d function redundantly as master regulators of the heat shock response (HSR) (Liu et al., 2011 *Plant Cell Enivronment* PMID: 21241330, Yoshida et al., 2011 *Mol Genet Genomics* PMID: 21931939, Liu et al., 2012 Plant Signal behaviour PMID: 22516818). Furthermore, studies have shown that both AtHSFA1a and AtHSFA1d can directly bind to the promoter elements of HSPs (Albertos et al., 2022, *EMBO J.*, PMID: 34981847; Luo et al., 2022, *Plant Communication*, PMID: 35927943). Additionally, several independent studies have highlighted the involvement of phytochrome-interacting factors (PIFs) and the auxin pathway in regulating the heat stress response (Gray et al., 1998, *PNAS*, PMID: 9618562; Sun et al., 2012, *PLOS Genetics*, PMID: 22479194; Koini et al., 2009, *Current Biology*, PMID: 19249207).

As mentioned earlier, since *atipk2βatipk2α^{kd}* plants are sensitive to heat stress, we speculated that the expression of these HSPs might be affected. To test this, we performed qPCR analyses and found that the expression of these HSPs was downregulated. Since the expression of HSPs is controlled by HSFs (as discussed above), we further speculated whether the activity of these HSFs in the knockdown lines was attenuated. Given the established role of IPMK proteins as transcriptional regulators in yeast and mammals, we examined the transcriptional activity of HSFs using luciferase assays and electrophoretic mobility shift assays (EMSA) in presence of IPMK-type proteins. As shown in Albertos et al., (2022) and Luo et al. (2022) [PMID: 35927943], *AtHSFA1a and d* directly binds to the promoter of *HSP70.3, HSP90* and *HSP18.2*. In our study, we successfully cloned and purified both *AtHSFA1a, HSFA1b* and *AtHSFA1d*. Since these three transcription factors function redundantly, and *AtHSFA1a/d* has already been characterized in Luo et al. (2022), we focused on *AtHSFA1b* for our experiments.

We hope this clarifies our rationale for selecting HSPs and focusing on AtHSFA1b in our study.

We agree with the reviewer that there could be many possible molecular scenarios through which AtIPK2 can control transcriptional activity of AtHSF1. While it is impossible to test all of them, we decided to explore the possibility whether IPK2 potentiates transcriptional activity of AtHSF1 through physical interaction considering that IPMK/IPK2 is already implicated in

transcriptional regulation in various eukaryotes through physical interaction. For instance, Kim et al., PNAS 2011, PMID: 24248338 (Fig. 3A of Kim et al.) demonstrated that IPMK binds to SRF, a mammalian transcription factor to potentiate its transcriptional activity. A study by Odom et al., Science 2000, PMID: 10720331 reported that “Ipk2 protein but not IP synthesis is needed to enable formation of ArgR-Mcm1 complexes on DNA promoter elements”. Furthermore, a recent study by Beon et al., eLife 2022 PMID: 35551737 reported that IPMK physically binds to SWI/SNF complex to control gene expression. Moreover, Ahn et al., NAR 2025 (PMID: 39777465) reported that IPMK potentiates the DNA-binding activity of the transcription factor SRF. Collectively, regulation of transcriptional activity of certain transcription factor by IPMK is well established in other eukaryotes. In this study, our findings suggest that IPK2/IPMK binds to HSF and promotes the transcriptional activity of AtHSF (Figure 9C-9L).

15. MAJOR: Figure 9 all panels. All gels are not very well resolved (protein-DNA complexes too close to the wells). Consider using a lower percentage of acrylamide to better separate the different molecular species. The EMSA assays require additional controls: Currently, there is no competitor DNA in the EMSA reaction. With the current experimental settings, any positively-charged DBD will bind to the negatively charged probe. Consider including a control experiment with a competitor DNA (like salmon sperm DNA) to establish the binding specificity of the probe. Along these lines, a mutated probe that abolishes binding should be tested alongside. Report molar concentrations for all proteins and probes concentrations. The current figure legend provides an interpretation rather than a description of the experiment. Line 459 change 'a short nucleotide fragment' for 'a short double stranded oligonucletotide'. Figure 9B, the enhancement of DNA binding in Figure 9B could be due to an artifact. Use another enzyme to check that the observed effect is specific to IPK2a (or to a domain of IPK2). Also, which amount of TF is used in Figure 9B? The legend for Figure 9D is incomplete. Figure 9E, If the hypothesis is that the enzyme affects the TF binding by interacting with it, then there should be a band for the TF alone and an upper band corresponding to the TF + AtIPK2a complex binding to the probe. But there seems to be only one band? The best way to address this point would be to identity missense mutations in the enzyme that impair the interaction of this mutant version with the TF and the probe in EMSA assays. The enhancement of DNA-binding in Figure 9E is not convincing. If the AtIPK2a enzyme indeed promotes DNA binding, then the intensity of the free probe should at least decrease. To aid comparison of Figure 9D and Figure 9E, the reactions should be performed with the same amounts of the HSF alone + probe (lane2). The percentage of bound probe should be similar but it is clearly not the same. For consistency, the interaction between the enzyme and the TF should also be tested for the Arabidopsis proteins, not only for the Marchantia orthologs in the Y2H assays, and possibly a second method (co-IP, in vitro pull down) could be used to substantiate this interaction.

We are thankful for all these important points. We have carefully gone through each comment and concern of the reviewer and have addressed them by conducting a series of new experiments. Our responses with revised experiments are outlined as below:

a) Regarding the concern that the complex is too close to the well: We agree with the reviewer’s observation. In response, we attempted running the samples on a lower percentage gel (4–5%); however, we observed a significant loss of sharpness in the bands, and the gel itself became too fragile to handle reliably. Therefore, we have used the same percentage of gel (6%) but have run the gel for a longer duration to improve resolution. The resolution is significantly improved with these changes, please see Fig. 9C to 9L.

b). Inclusion of unlabelled oligo as competitor in EMSA: As suggested by the reviewer, we have performed the EMSA experiment using an unlabelled oligonucleotide as a competitor. The corresponding data has been incorporated as Fig. 9E and 9J.

c). Use of mutant probe lacking the canonical HSE element: As pointed out by the reviewer, we have carried out EMSA using a mutant probe that lacks the canonical HSE (GAANNTTC) sequence. Our results show that the binding of AtHSFA1b and MpHSFB1 is specific to the HSE element. This data has been included as Fig. 9D and 9I.

d). *Inclusion of protein molar concentrations in figure legends:* Following the reviewer's suggestion, we have included the molar concentrations of the proteins used in the EMSA experiments in the legend of *Figure 9*. Additionally, we have revised the figure legend to provide a more detailed description of the experimental conditions.

e). *Correction of statement:* We have revised the phrase "a short nucleotide fragment" to "a short double-stranded oligonucleotide". Thank you for the suggestion.

f). Suggestion to use another protein rather than AtIPK2. To further strengthen our findings, we included MBP as a control. We observed that the addition of MBP had no effect on the DNA-binding affinity of AtHSFA1b or MpHSFB1. This supports the conclusion that the enhancement of HSFB1 DNA-binding activity upon addition of AtIPK2b is specific and not an artifact. Please see the corresponding data in the revised manuscript Fig. 9G and 9L.

g). Completion of Figure legend of 9D. We have revised the figure legend for *Figure 9D* to include all relevant experimental details.

h). Concern regarding complex formation. We agree with the reviewer that if IPMK directly binds to the DNA–TF complex, a third band corresponding to the ternary complex may be expected. However, the absence of such a band does not exclude the possibility of this interaction. The observed increase in the DNA–HSFB1 complex, accompanied by a corresponding decrease in the free probe upon addition of IPMK, suggests that IPMK enhances the DNA-binding activity of HSFB1. While generating a missense mutant after mapping the IPMK/IPK2 binding site to TF would provide further insights, these experiments might require year-long study and we feel this is beyond the scope of this study. In the current study, we have used a series of control as instructed by the referee that clarifies the concerns raised by the referee as evident by the experimental data.

i) HSF and AtIPK2/IPMK interaction. The referee correctly pointed out that TF and the enzyme should interact physically given that addition of enzymes potentiates the DNA-binding activity of TF. As instructed by the referee, we show that AtIPK2 and AtHSF interact physically using independent assays such as Y2H and BiFC assay. Similarly, we found that MpIPMK interacts physically with MpHSF using both Y2H and BiFC assays. The corresponding data have been incorporated as Fig. 9A, 9B and Supplementary Figure 12B.

In summary, we have completely revised the Fig. 9 by performing a series of experiments as instructed by the referee that were instrumental to strengthen our findings and conclusion.
