# Supplementary material for: Conservation of heat stress acclimation by the IPK2-type kinases that control the synthesis of the inositol pyrophosphate 4/6-InsP7 in land plants
Source: PLoS Genet. 2025 Sep 11;21(9):e1011838. doi: 10.1371/journal.pgen.1011838 (PMC12445474; doi:10.1371/journal.pgen.1011838)
Supplement: Attachment — Submitted filename: Response to Reviewer Comments_July 2025.pdf [file pgen.1011838.s022.pdf]

Reviewer #1: All my questions have been addressed

We are grateful to the reviewer for the constructive feedback that have significantly improvised our manuscript. We sincerely appreciate the reviewer's valuable time and support throughout the review process.

Reviewer #2: The revised manuscript by Yadav et al. represents a substantial improvement and now more accurately reflects the experimental work presented. The EMSA assays have been refined, and the discussion surrounding the $InsP_3$ versus $InsP_6$ kinase activity has become more balanced.

We are grateful to the reviewer for the constructive feedback that have significantly improvised our manuscript. We sincerely appreciate the reviewer's valuable time and support throughout the review process.

Figure 1C: The ITC experiments are now supported by appropriate control experiments. However, the observed stoichiometries do not align with a simple 1:1 enzyme–substrate model, likely due to the small differential power signal and considerable data noise. While this does not undermine the experiment, it suggests that the derived dissociation constants may lack accuracy. I recommend leaving the experiments as they are.

Thank you for the recommendation. Following the reviewer suggestion, we have retained the ITC experimental findings as they are. As discussed in the previously submitted version of our manuscript, our ITC analyses revealed that IPK2 proteins bind to $InsP_6$ with greater affinity, likely due to the crowded negative charges of $InsP_6$. Based on these findings, we did not claim that $InsP_6$ is a preferred substrate over $InsP_3$. To address this point further, we determined kinetic analyses as instructed by the reviewer, and the findings were incorporated in the previously submitted manuscript.

Lines 198–200: The current reasoning is convoluted — implying that low activity toward a substrate confirms its correctness is not convincing. It would be more straightforward to state that AtIPK2 is a relatively inefficient $InsP_6$ kinase, and that $InsP_3$ appears to be the preferred in vitro substrate.

We agree with the changes. Accordingly, we have revised the text to reflect the reviewer suggestions that $InsP_3$ is a preferred *in vitro* substrate of AtIPK2. Please see line 194 to 197.
